# Multistage targeting and dual inhibiting strategies based on bioengineered tumor matrix microenvironment‐mediated protein nanocages for enhancing cancer biotherapy

**DOI:** 10.1002/btm2.10290

**Published:** 2022-01-05

**Authors:** Fabiao Hu, Changping Deng, Yiwen Zhou, Yuping Liu, Tong Zhang, Peiwen Zhang, Zhangting Zhao, Hui Miao, Wenyun Zheng, Wenliang Zhang, Meiyan Wang, Xingyuan Ma

**Affiliations:** ^1^ State Key Laboratory of Bioreactor Engineering East China University of Science and Technology Shanghai China; ^2^ Shanghai Key Laboratory of New Drug Design School of Pharmacy, East China University of Science and Technology Shanghai China; ^3^ Center of Translational Biomedical Research University of North Carolina at Greensboro Greensboro North Carolina USA; ^4^ Synthetic Biology and Biomedical Engineering Laboratory, Biomedical Synthetic Biology, Research Center, Shanghai Key Laboratory of Regulatory Biology, Institute of Biomedical, Sciences and School of Life Sciences East China Normal University Shanghai China

**Keywords:** cancer precision therapy, ferritin heavy chain nanocages, matrix metalloproteinase‐2, survivin, transcription and protein levels

## Abstract

Regulation of the apoptotic pathway plays a critical role in inducing tumor cell death and circumventing drug resistance. Survivin protein is the strongest inhibitor of apoptosis found so far. It is highly expressed in several cancers and is a promising target for cancer therapy. However, clinical applications are limited by incomplete inhibition of survivin expression. Here, we present a novel strategy that extended the release of YM155 (an effective survivin inhibitor that works by inhibiting the activity of survivin promoter) and TATm‐survivin (T34A) (TmSm) protein (survivin protein mutant with penetrating peptide, a potential anticancer protein therapeutic) via tumor matrix microenvironment‐mediated ferritin heavy chain nanocages (FTH1 NCs), enabling significant inhibition of survivin activity at both transcript and protein levels. FTS (FTH1‐matrix metalloproteinase‐2‐TmSm)/YM155 NC synthesis was easily scaled up, and these NCs could sequentially release TmSm protein through matrix metalloproteinase‐2 and promote YM155 to enter the nucleus via transferrin receptor 1 (TfR1) binding, which increased the cytotoxicity and apoptosis of Capan‐2 and A549 cells compared to that with individual drugs. Moreover, FTS/YM155 NCs enhanced drug accumulation at tumor sites and had a higher tumor inhibition rate (88.86%) than the compounds alone in A549 tumor‐bearing mice. In addition, FTS/YM155 NCs exerted significant survivin downregulation (4.43‐fold) and caspase‐3 upregulation (4.31‐fold) and showed better therapeutic outcomes without inducing organ injury, which highlights their promising future clinical application in precision therapy. This tumor microenvironment‐responsive platform could be harnessed to develop an effective therapy via multilevel inhibition of cancer targets.

## 1 INTRODUCTION

Evasion of cell apoptosis is a crucial cause of resistance to traditional chemotherapy and radiotherapy.^1^, ^2^ Therefore, the inhibitor of apoptosis proteins (IAPs) have been considered potential targets for cancer therapy.^3^ One member of the smallest endogenous IAP family, survivin, has attracted attention as a potential cancer‐specific target.^4^, ^5^ Currently, various strategies have been developed to downregulate or block survivin expression to enhance apoptosis and attenuate tumor growth using promoter inhibitors, antisense oligonucleotides, ribozymes, microRNAs, and small interfering RNAs (siRNAs); these molecules target survivin or antagonize the activation and stabilization of survivin by regulating survivin‐suppressing molecules.^6^, ^7^

Despite efforts to inhibit survivin at the transcription or protein level in preclinical and clinical research,^7^, ^8^ the potential clinical application is limited by challenges related to the poor efficacy of single‐level survivin inhibition or adverse effects caused by systemic administration.^9^, ^10^ Therefore, there is an urgent need to target survivin at both the transcriptional and protein levels to improve cancer therapy. Sepantronium bromide (YM155), a novel survivin inhibitor, can suppress survivin expression at the transcription level by binding to the transcription factor Sp1 in many cancer cells.^11^ In our previous studies, TATm‐survivin (T34A) protein (TmSm) is the most effective survivin mutant. It promotes the apoptosis of breast, pancreatic, and liver cancer cells, and significantly inhibits the tumor growth of breast cancer tumor‐bearing mice.^12^, ^13^ In this regard, the combined cancer therapy of YM155 and TmSm is an attractive option for synergistically enhancing anticancer activity. However, although it has good clinical treatment prospects, the optimal anticancer effect cannot be achieved due to the shortcomings of traditional administration, including the short half‐life of free protein and small molecule drugs, inefficient delivery, and poor bioavailability in vivo.^14^, ^15^ Self‐assembled protein nanocages (NCs) represent a class of nanoscale platforms that hold much promise for co‐incorporating small molecules and proteins to improve anticancer therapy and overcome the existing limitations.^16^, ^17^

Recently, naturally produced human ferritin heavy chain nanocages (FTH1 NCs) assembled from 24 subunits were discovered as protein‐based nanoparticles that offer advantages over synthetic polymers in terms of biostability, biocompatibility, and biodegradability for delivering diverse imaging agents and protein drugs^18^, ^19^, ^20^ and encapsulating various chemotherapeutic drugs, such as curcumin, doxorubicin (DOX), and paclitaxel.^21^, ^22^, ^23^ In addition, FTH1 NCs can specifically bind to cancer cells by interacting with transferrin receptor 1 (TfR1).^21^, ^24^ However, TfR1 is broadly expressed in osteoclasts and activated lymphocytes and has a risk of off‐target toxicity. Therefore, strategies for shielding the exposured ligands on the NC surface need to be developed. Matrix metalloproteinase‐2 (MMP‐2) is a secreted enzyme that promotes tumorigenesis and development. In addition, MMP‐2 is overexpressed in malignant tumor tissues compared with blood and benign tissues and is also used as a biomarker for cancer therapy and diagnosis.^25^ In recent studies, MMP‐2 has been used as a tumoral stimulus for tumor‐targeted drug release.^26^

**FIGURE 1:**
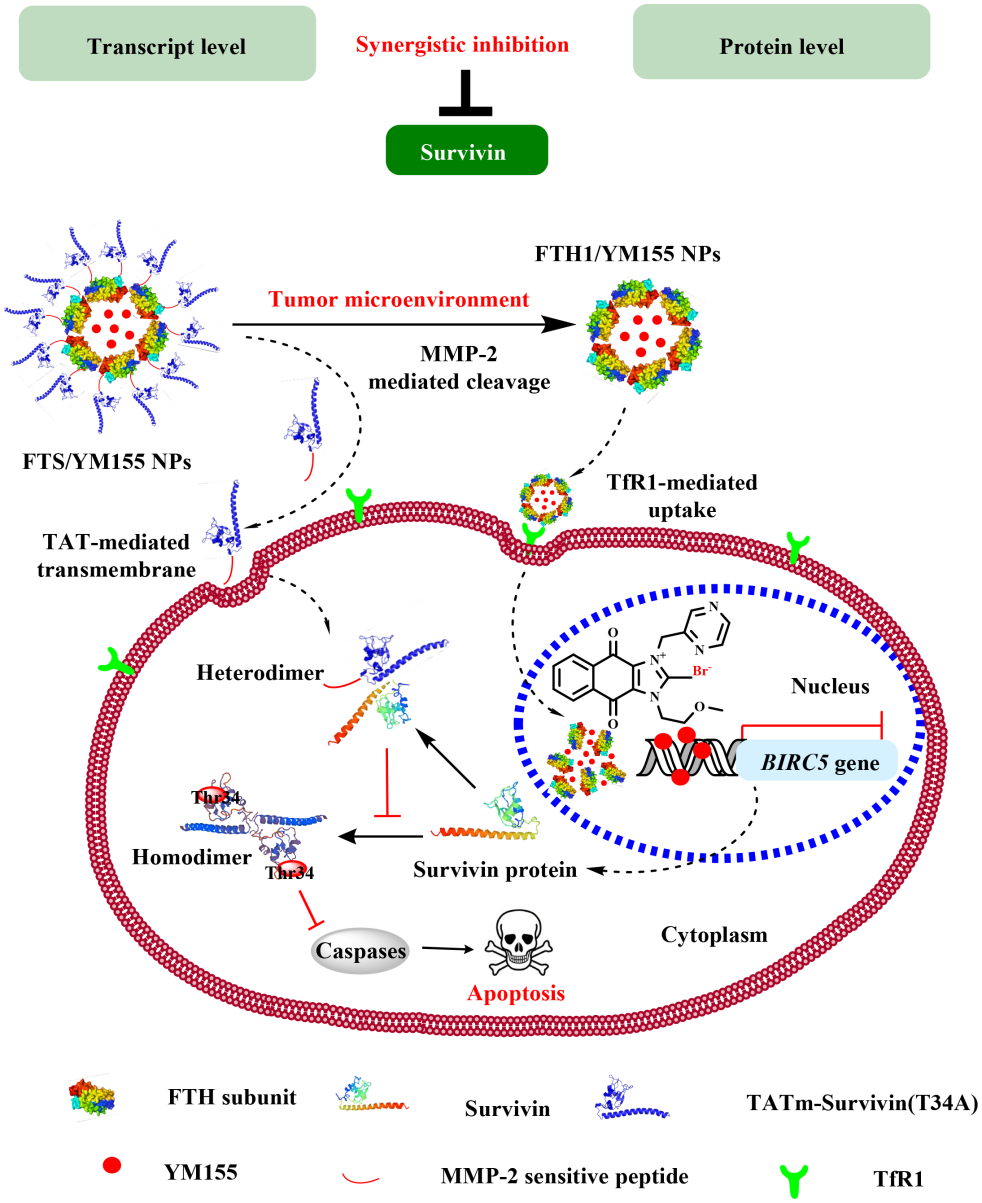
Schematic diagram illustrating the synthesis and delivery mechanism of FTS/YM155 NCs. TmSm was genetically fused to the C‐terminus of FTH1 gene through MMP‐2 sensitive peptide and YM155 was encapsulated in the hollow nanocage (NC) of FTS NCs by urea‐mediated disassembly/assembly. The developed FTS/YM155 NCs were accumulated in the tumor site after intravenous injection. FTS/YM155 NCs could sequentially release TmSm protein in tumor microenvironment and YM155 into cancer cells via responding to enriched MMP‐2 and binding of TfR1. TmSm was delivered to the cytoplasm through TAT peptide and formed a heterodimer with wild‐type survivin, which could be degraded by the ubiquitination pathway. YM155 was released to the nucleus to inhibit the transcription expression of survivin. Overall, FTS/YM155 NCs could synergistically inhibit survivin at the transcription and protein levels. MMP‐2, matrix metalloproteinase‐2; TfR1, transferrin receptor 1

Here, we developed MMP‐2‐sensitive FTH1 NCs—specifically TmSm‐modified FTH1 NCs carrying YM155 (FTS/YM155 NCs)—for the codelivery of YM155 and TmSm to synergistically inhibit survivin activity at both the transcript and protein levels (Figure 1) at a tumor‐specific site. In this study, TmSm was fused to the C‐terminus of the *FTH1* gene via an MMP‐2‐sensitive peptide (P‐L‐G‐L‐A‐G), which is responsive to the tumor microenvironment for site‐specific release of the TmSm protein. Moreover, YM155 encapsulated in hollow NCs by the disassembly/reassembly method enters the cellular nucleus by TfR1 binding, enabling the inhibition of survivin transcription. Also, we selected three cell lines for further experiments, including A549 (human nonsmall cell lung cancer cells), Capan‐2 (human pancreatic cancer cells), and HUVEC (human umbilical vein endothelial cells). We examined the anticancer effect of FTS/YM155 NCs *in vitro* and in vivo and found that this strategy could efficiently deliver high doses of therapeutic drugs to improve therapeutic outcomes and induce their selective accumulation in cancer cells in a tumor microenvironment‐stimuli responsive manner. The high performance and safety of the NCs make this platform to a promising modality for cancer treatment in the clinic. This approach can therefore be expanded to different cancer biomarkers, which may in turn facilitate the clinical progress of precision therapy.

## 2 MATERIALS AND METHODS

### 2.1 Materials

YM155 (CAS Number: 781661‐94‐7) and DOX (CAS Number: 25316‐40‐9) were supplied by Aladdin. Hoechst33342 (CAS Number: C0031) and cyanine (CAS Number: 1562‐85‐2) were supplied by Solarbio Life Sciences. Other chemicals and reagents were analytical grade. A549, Capan‐2, and HUVEC cells were obtained from the Type Culture Collection Committee of Chinese Academy of Sciences.

### 2.2 Biosynthesis and purification of FTH1 and FTS proteins

The expressed plasmid harboring FTH1 or FTS gene was constructed according to the previously described procedure.^27^ Briefly, human *FTH1* gene was cloned using molecular cloning techniques (Sino Biological Inc.) and *TmSm* gene was derived from our previous studies.^13^ The sequences of flexible peptide (G‐G‐G‐G‐S‐G‐G‐G‐G‐S) and MMP‐2 sensitive peptide (G‐P‐L‐G‐L‐A‐G) were introduced between *TmSm* and *FTH1* genes by overlapping extension polymerase chain reaction (PCR) to obtain FTH1‐Linker‐MMP2‐TmSm (FTS). The PCR products were ligated to the pET‐24a(+) plasmid and transformed into *Escherichia coli* BL21 *(DE3)* (Novagen). Moreover, three‐dimensional (3D) structure of protein was modeled using I‐TASSER software (http://zhanglab.ccmb.med.umich.edu/I-TASSER/).

The recombinant proteins (FTH1 and FTS) expression and purification were carried out according to the previously described method with moderate adjustments.^23^ FTH1 protein was expressed in the supernatant, whereas FTS protein mainly existed in inclusion bodies. The supernatant was heated at 60°C for 10 min to precipitate heat‐sensitive proteins. Inclusion bodies were dissolved in Buffer A (20 mM phosphate buffer [PB], 8 M urea, pH 7.1) followed by washing three times with Buffer B (20 mM PB, 0.5 mM NaCl, 2 M urea, and 0.5% Triton X‐100, pH 7.1). Afterward, the heated supernatants or denatured proteins were purified by the diethylaminoethyl (DEAE) sepharose column (GE Healthcare), respectively. For protein purified from inclusion bodies, the eluted fraction from denatured protein was refolded by sequentially dialyzing in Buffer C (20 mM PB, 50 mM NaCl, 10% glycerol, pH 7.4) containing 4, 2, 1, or 0 M of urea. For protein purified from supernatant, the eluted fraction from the heated supernatant was dialysis using Buffer D (10 mM phosphate‐buffered solution [PBS], pH 7.4). The purified protein was analyzed using sodium dodecyl sulfate–polyacrylamide gel electrophoresis (SDS‐PAGE), and its concentration was measured by Bradford assay.

### 2.3 Preparation of YM155‐loaded NCs

FTS/YM155 NCs and FTH1/YM155 NCs were prepared by urea‐mediated and pH‐mediated disassembly/assembly methods with minor modification, respectively.^21^ Briefly, YM155 dissolved in PBS solution (1 mM) was added to FTS NCs or FTH1 NCs dissolved in PBS solution (2 μM, 5 mL) at NCs/YM155 molar ratio of 1:200. Among them, FTS NCs from mixed solutions were disassembled into subunits by 8 M urea, stirred for 60 min, and sequentially dialyzed with Buffer C. FTH1 NCs from mixed solutions were adjusted to pH 2.0 by 0.1 M HCl to disassemble into subunits, and then increased to pH 7.4 with 1.0 M NaOH after stirring for 60 min, and dialyzed with Buffer D. After dialysis and centrifugation, the supernatants were concentrated with30 kDa Amicon Ultra device (ACS503002) (Millipore) and filtered by 0.22‐μm sterile filter (F513163‐0001) (Sangon Biotech). DOX‐loaded NCs and cyanine‐loaded NCs were fabricated in a similar way.

### 2.4 Characterization of FTS/YM155 NCs

The morphology of FTH1‐based NCs was characterized using transmission electron microscopy (TEM; JEOL). Briefly, the samples (0.1 mg/ml, 10 μl) were dropped on carbon‐coated copper grids, and negatively stained by 2% phosphotungstic acid. TEM micrographs were imaged at 120 kV. The zeta potential and particle size of NCs were detected by dynamic light scattering (DLS) (Malvern) at 25°C. Full wavelength scanning of YM155, FTS NCs, and YM155/FTS NCs were measured by ultraviolet to visible (UV–Vis) spectrophotometer (Hitachi) at 250–500 nm. Protein secondary structure was assessed using circular dichroism (CD) spectrometer (Applied Photophysics). The CD spectra of samples (0.1 mg/ml in PBS) were recorded from 260 to 200 nm in a 1 mm quartz cell at 25°C. YM155 contents in FTS/YM155 NCs and FTH1/YM155 NCs were measured by UV–Vis spectrophotometer.^23^ Protein concentrations of FTS/YM155 NCs and FTH1/YM155 NCs were determined by Bradford assay (C503031‐1000) (Sangon Biotech). The protein sample (0.1 mg/ml) was adjusted to pH 2.0 to release YM155, and YM155 content was detected using a UV–Vis spectrophotometer at 349 nm. The drug loading content (LC) and encapsulation efficiency (EE) were calculated by the following formulas, respectively.

$$\mathrm{LC}\;\left(\%\right)=\left(\text{weight of}\;\mathrm{YM}155\;\text{encapsulated in}\;\mathrm{NCs}/\text{weight of}\;\mathrm{NCs}\right)\times 100\%$$

$$\mathrm{EE}\;\left(\%\right)=\left(\text{amount of}\;\mathrm{YM}155\;\text{encapsulated in}\;\mathrm{NCs}/\text{total amount of}\;\mathrm{YM}155\right)\times 100\%$$

### 2.5 In vitro drug release profile and stability of FTS/YM155 NCs

The drug release of NCs was investigated by dialysis method.^28^ Briefly, FTS/YM155 NCs and FTH1/YM155 NCs (10 mg) were loaded into dialysis membrane (MWCO of 7 kDa), dialyzed against 50 ml of PBS (pH 7.4 and 5.0), and shaken at 100 rpm at 37°C. The sample (1 ml) was collected at appropriate intervals (1, 2, 4, 8, 12, 18, 24, 36, 48, and 72 h) and immediately added an equal amount of fresh PBS. The centrifuged samples were measured using UV–Vis spectrophotometer at 349 nm. For stability analysis, nanoparticle samples (1 mg/ml) were dispersed in PBS or 50% (vol/vol) fetal bovine serum (FBS) and shaken in at 100 rpm at 37°C. The particle sizes of NCs were detected at 0 and 24 h using DLS to assess the stability of NCs.

### 2.6 Cell culture

Capan‐2, A549, and HUVEC cells were, respectively, cultured in RPMI 1640 (CAS Number: 11875093) and Dulbecco's modified Eagle medium (DMEM) medium (CAS Number: 11995040) (Gibco) supplemented with 10% FBS (CAS Number: 10099141C) (Gibco) and 1% penicillin/streptomycin solution (CAS Number: 15070063) (Gibco). All cells were maintained at 37°C in 5% CO_2_ atmosphere conditions.

### 2.7 Gelatin zymography

The MMP‐2 enzymatic activity was detected by gelatin zymography as previously described method.^26^ Briefly, A549 and Capan‐2 cells with 80%–90% confluence were cultured in RPMI 1640 minimal medium. After 48 h incubation, the conditioned media were concentrated and quantified by bicinchoninic acid (BCA) kit (C503021‐0500) (Sangon Biotech). Subsequently, protein samples were added onto 15% Native‐PAGE gels containing 0.1% (wt/vol) gelatin. After protein separation, the gel was renatured in activation buffer (50 mM Tris–HCl, 200 mM ZnCl_2_, 5 mM CaCl_2_, and 0.05% Brij‐35, pH 7.5) for 22 h at 37°C and stained with 0.25% coomassie blue. The intensity of bands represented gelatinolytic activity quantified by ImageJ (National Institutes of Health).

### 2.8 Cleavage and kinetic profile of FTS NCs

The MMP‐2‐mediated cleavage of FTS NCs was investigated in conditioned medium derived from A549 cells using SDS‐PAGE electrophoresis. FTS NCs (4 μg) were incubated with the A549‐conditioned medium for 3 and 6 h at 37°C, respectively. The cleavage efficiency of FTS NCs was measured via SDS‐PAGE electrophoresis. For cleavage kinetic analysis of FTS NCs, A549 cells were cultured at 2 × 10^6^ cells per well in sixwell plates for 24 h before treatment with FTS NCs (0.2 mg/ml). At scheduled times (1, 2, 4, 6, 12, 24, and 48 h), 60 μl of cell culture medium was taken out and terminated the reaction with 20 μl of Native‐PAGE loading buffer (9175) (Takara). The obtained samples were separated by Native‐PAGE electrophoresis to analyze the MMP‐2‐catalyzed kinetics.

### 2.9 Cellular uptake

A549 and Capan‐2 cells (5 × 10^4^ cells per well) were cultured in confocal Petri dish (NEST) for 24 h and then incubated with free DOX, FTH1/DOX NCs, and FTS/DOX NCs at a concentration of 0.33 μM (equivalent to 0.60 μM DOX) for 6 and 12 h, respectively. The washed cells were fixed by 4% paraformaldehyde and stained by Hoechst 33342. The fluorescent images were taken by confocal laser‐scanning microscope (CLSM) (Nikon) using Tetramethylrhodamine and 4′,6‐diamidino‐2‐phenylindole channels. For quantitative cellular uptake, the cells were seeded into sixwell plates and treated with free DOX, FTH1/DOX NCs, and FTS/DOX NCs (equivalent to 0.60 μM DOX) for 6 and 12 h, respectively. Afterwards, the treated cells were digested, washed and resuspended in PBS. The resuspended cells were analyzed using flow cytometry (FCM) (Becton Dickinson) equipped for PE.

Antibody blocking experiments were used to confirm that FTH1 NCs could be uptake by cancer cells through the binding of TfR1. FTH1/DOX NCs and FTS/DOX NCs (0.33 μM) were added to A549 and Capan‐2 cells, and incubated with anti‐TfR1 mAb (3.3 μM) for 12 h. CLSM and FCM were used to analyze the fluorescence intensity of cells incubated in the presence or absence of TfR1 mAb (Cat No: 66180‐1‐Ig) (Proteintech).

### 2.10 In vitro cytotoxicity assay

The cytotoxicity of FTS/YM155 NCs against cancer cells was assessed by MTT method. Capan‐2 and A549 cells were seeded overnight in a 96‐well plate at a density of 5 × 10^3^ cells per well. Cells were incubated for 24 and 48 h in DMEM containing different concentrations of FTS NCs and FTS/YM155 NCs (0.04, 0.08, 0.17, 0.25, and 0.33 μM), respectively. Thereafter, each well was added MTT solution (5 mg/ml, 20 μl) and incubated for an additional 4 h. The formazan dissolved in dimethyl sulfoxide was used to measure the absorbance at 490 nm using a microplate reader (Biotek Instruments Inc). Cell viability and half‐maximal inhibitory concentrations (IC_50_) were calculated. Based on the IC_50_ value of FTS/YM155 NCs, varying concentrations of drugs treated A549 and Capan‐2 cells for 24 and 48 h, respectively. The cytotoxicity of TmSm (0, 1.04, 2.08, 4.17, 6.25, and 8.33 μM) and YM155 (0, 0.001, 0.01, 0.05, 0.1, and 0.2 μM) against both cells were performed as described above.

### 2.11 Annexin V‐FITC apoptosis detection

The apoptosis of cancer cells was determined by Annexin V‐FITC detection kit (C1062S) (Beyotime Biotech). Briefly, A549 and Capan‐2 cells were cultured overnight in a sixwell plate at a density of 2 × 10^5^ cells per well and treated in varying concentrations of drugs for 48 h. The final concentrations of TmSm and YM155 were kept at 2 and 0.15 μM, respectively. The harvested cells were resuspended in 200 μl of binding buffer and stained with Annexin V‐FITC and propidium iodide (PI) at 25°C. The stained cells were analyzed using FCM equipped for FITC and PI.

### 2.12 Hemolysis assay

The biocompatibility of NCs was evaluated by hemolysis assay.^21^ The red blood cells (RBC) were washed and diluted 10‐fold with washing buffer (PBS containing 25 U/ml of heparin). 200 μl of diluted RBC suspension was mixed with drug solutions (800 μl) at various concentrations (0.04, 0.08, 0.17, 0.25, and 0.33 μM). Ultrapure water and PBS were set as positive and negative control groups, respectively. The mixtures were incubated for 3 h at 37°C and centrifuged (12,000 rpm, 15 min) to collect the supernatant. The absorbance of hemoglobin in the supernatant was detected using UV–Vis spectrophotometer at 541 nm. The hemolysis rate (HR) of different drug treated groups was calculated by the formula

$$\mathrm{HR}\;\left(\%\right)=\left({\mathrm{OD}}_{t}-{\mathrm{OD}}_{n}\right)/\left({\mathrm{OD}}_{p}-{\mathrm{OD}}_{n}\right)\times 100\%$$

wherein, OD_t_ refers to the absorbance of the drug group, OD_n_ and OD_p_ refer to the absorbance of the negative and positive controls, respectively.

### 2.13 In vivo biodistribution and anticancer effects evaluation

For biodistribution, 1 × 10^7^ A549 cells were harvested and injected subcutaneously into the left hind limb of the BALB/c nude mice (Shanghai SLAC Laboratory Animal Co., Ltd). Free cyanine and cyanine‐loaded NCs (100 μl, 5 mg/kg cyanine equivalents) were injected into mice via the tail vein when the tumors of A549 tumor‐bearing mice reached about 200 mm^3^. The fluorescent images were taken at 0.5, 4, and 24 h after injection by multispectral imaging system (KODAK) with the excitation and emission wavelength at 740 and 830 nm. After imaging, mice were humanely sacrificed and their major organs and tumors were collected followed by fluorescence imaging.

In an antitumor efficacy study, when tumors reached approximately 150 mm^3^, A549 tumor‐bearing mice were randomized into eight groups (*n* = 5). The different concentration of NCs (5 mg/kg TmSm and 0.01 mg/kg YM155 equivalents) were intravenously injected via tail vein every 3‐day for 12 days. The tumor size and body weight of the mice were recorded every 3‐day for 12 days. After treatment, the tumors were weighed and photographed. The tumor volumes were calculated using the formula: tumor volume = [length of tumor × (width of tumor)^2^]/2. The major organs were fixed with 4% paraformaldehyde for paraffin slicing and stained with hematoxylin and eosin for histological examination. The expression of caspase‐3 and survivin in tumor tissues was detected by immunohistochemistry. The survival rate of the mice was recorded every 3‐day and analyzed by Kaplan–Meier survival curve. Mice were statistically regarded as imminent deaths during treatment when mice naturally died or tumor volume exceeded 2000 mm^3^.^29^

### 2.14 Quantitative real‐time PCR analysis

RNA of cells or tissues was isolated by the EZ‐press Cell to cDNA Kit PLUS (CB05833067) (EZBioscience). The quantitative real‐time PCR (qRT‐PCR) reactions were conducted on the Bio‐Rad CFX96 qRT‐PCR instrument (Bio‐Rad) by a SYBR Green qRT‐PCR Master Mix (RR036A) (Takara Biomedical). Program for qRT‐PCR amplifications were as follows: 95°C for 5 s, followed by 40 cycles at 95 °C for 5 s, 55°C for 10 s, and 72°C for 20 s. The qRT‐PCR primer was shown in Table S1.

### 2.15 Western blot analysis

Cells or tissues were lysed in RIPA buffer (9806S) (Cell Signaling Technology) and protein concentration in samples was quantified by BCA kit (Sangon Biotech). Proteins (30 μg) were separated on a 12% SDS‐PAGE and electrotransferred to the treated polyvinyl difluoride membrane (88585) (Thermo Fisher Scientific). After blocking, the membranes were incubated with the primary antibodies against surviving (Cat No: 66495‐1‐Ig), MMP‐2 (Cat No: 66366‐1‐Ig), TfR1 (Cat No: 66180‐1‐Ig), caspase‐3 (Cat No: 66470‐2‐Ig), and β‐actin (Cat No: 66009‐1‐Ig) (Proteintech) overnight at 4°C, then washed thrice with PBS and incubated with the horseradish peroxidase‐conjugated goat anti‐rabbit (Cat No: SA00001‐2) or anti‐mouse IgG (Cat No: SA00001‐1) (Proteintech). Protein bands were detected by a chemiluminescence kit (Order NO: D601039) (Sangon Biotech) and quantitated by ImageJ software.

### 2.16 Ethics

All experiments involving animals were approved by Chinese legislation on the use and care of research animals (Document No. 55, 2001) and controlled by the animal ethics committee of East China University of Science and Technology (REC No. 20181223). After the experiments, all mice were euthanized.

### 2.17 Statistical analysis

All experiments were performed in triplicate unless otherwise stated, and the results were expressed as mean ± *SD*. Statistical significance values were evaluated through one‐way analysis of variance test with posthoc contrasts by Student–Newman–Keuls test, or part of the data were conducted by Student's *t*‐test, using SPSS 22.0 software (IBM) for evaluation. Differences were considered statistically significant at *p* < 0.05 (*), very significant at *p* < 0.01 (**), and extremely significant at *p* < 0.001 (***).

## 3 RESULTS

### 3.1 Biosynthesis and purification of FTS and FTH1 proteins

To prevent nonspecific cell penetration of TmSm protein during intravenous delivery,^30^ the TmSm sequence was fused to the C‐terminus of *FTH1* gene. MMP is reported to release depots that dynamically respond to their environment.^31^, ^32^ In this study, the MMP‐2‐sensitive peptide and flexible peptide were inserted between the FTH1 and TmSm proteins, enabling the release of the TmSm protein at the tumor site (Figure S1a–c). Meanwhile, FTS and FTH1 proteins were expressed (Figure S2a–c), purified using DEAE Sepharose chromatography, and detected by SDS‐PAGE (Figure S3a,b). In addition, the correct spatial structure was confirmed using 3D structure simulation, and the purity of the corresponding protein was calculated (Figure S4a,b). Interestingly, the renaturation rate of the FTS protein was 1.91‐fold that of the TmSm protein, indicating that fusion of TmSm and FTH1 significantly increased the renaturation rate of the TmSm protein (Figure S4c).

### 3.2 Preparation and characterization of YM155‐loaded NCs

**FIGURE 2:**
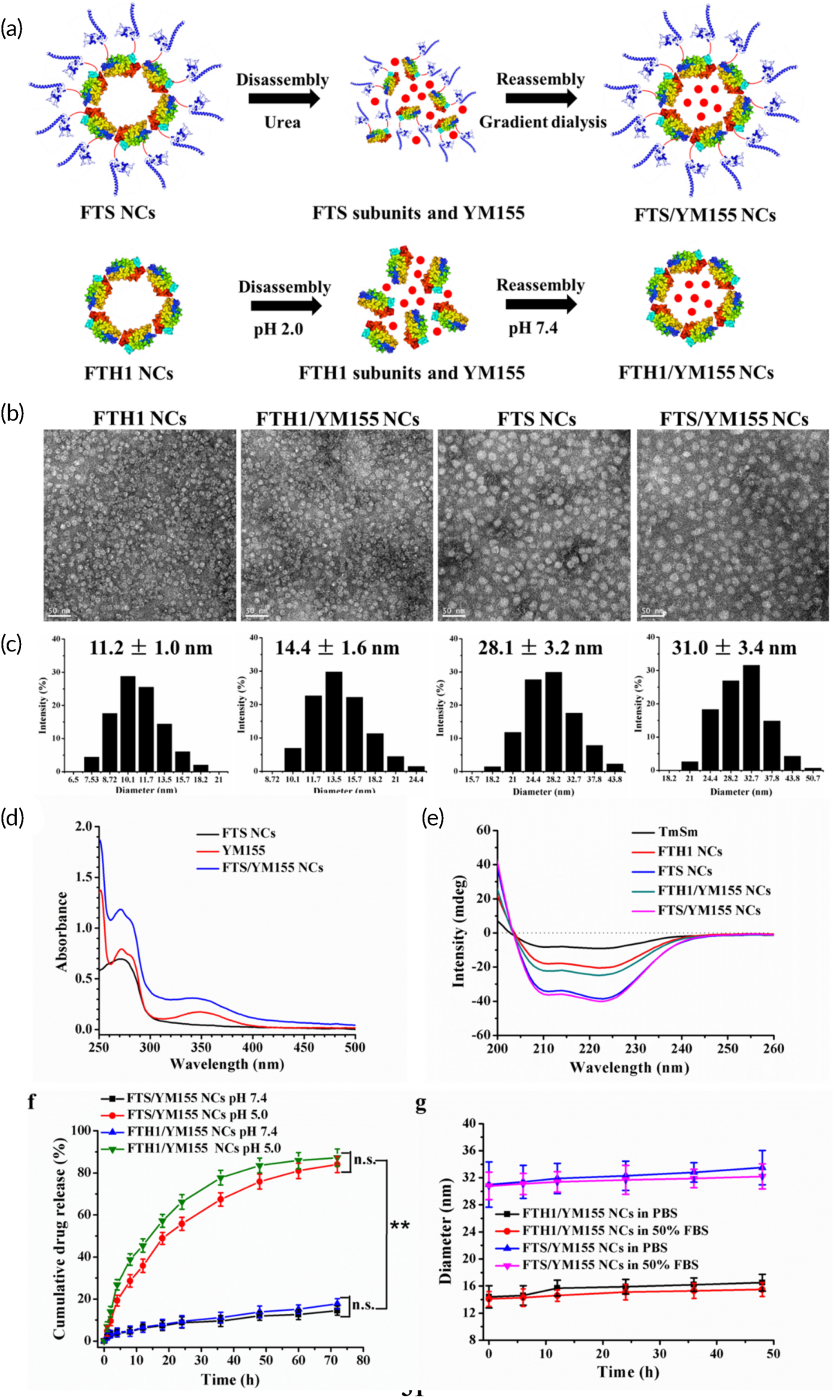
Preparation and physicochemical characterization of FTS/YM155 NCs. (a) Schematic diagram of preparation of FTS/YM155 NCs and FTH1/YM155 NCs. (b) The morphology and (c) particle size distribution of NCs were measured using TEM and DLS (Scale bar = 50 nm). (d) UV–Vis absorption spectrum and (e) CD spectra of NCs. (f) In vitro drug release profiles of FTH1/YM155 NCs and FTS/YM155 NCs in PBS (pH 7.4 and 5.0). (g) Stability assessment of NCs in PBS and 50% FBS at 37°C for 48 h of incubation. Data were expressed as mean ± *SD* (*n* = 3). Statistical analysis was by the two‐tailed *t*‐test; **p < 0.01. CD, circular dichroism; DLS, dynamic light scattering; NC, nanocage; n.s., not significantly different; PBS, phosphate‐buffered solution; TEM, transmission electron microscopy; UV–Vis, ultraviolet to visible

FTH1 NCs with hollow NCs (8 μm) and reversible assembly characteristics provided the nanoscale scaffolds for encapsulating small molecule drugs.^33^ To establish NCs loaded with the survivin inhibitor YM155, YM155‐loaded FTS NCs and FTH1 NCs (FTS/YM155 NCs and FTH1/YM155 NCs, respectively) were prepared by urea‐mediated and pH‐mediated disassembly/assembly methods (Figure 2a) due to FTH1 and FTS proteins expressed as soluble and inclusion bodies. Moreover, FTH1 NCs could disassemble into protein subunits in acidic medium (pH 2.0) and release the encapsulated molecules because YM155 was stable in acidic and neutral media, while it was unstable in alkaline medium (Figure S5a,b). Then, the optimal TmSm/YM155 molar ratio for synthesizing FTS/YM155 NCs was determined. When protein and YM155 were added to NCs at a feed molar ratio of 1:10, the YM155/NP molar ratios in FTH1/YM155 NCs and FTS/YM155 NCs were 1.6 and 1.8, respectively (Figure S5c); these formulations had similar cytotoxicity to cancer cells (Figure S5d). Moreover, the EE and LC of the FTS/YM155 NCs were 19.34 ± 1.21% and 0.08 ± 0.04%, respectively (Table 1).

**TABLE 1:** Characterization of FTS/YM155 NCs

	FTH1 NCs	FTS NCs	FTH1/YM155 NCs	FTS/YM155 NCs
Molecular weight (kD)	534.96	996.41	ND	ND
Diameter (nm)	11.2 ± 1.0	28.1 ± 3.2	14.4 ± 1.6	31.0 ± 3.4
Polydispersity index	0.172 ± 0.013	0.212 ± 0.016	0.196 ± 0.018	0.271 ± 0.026
Zeta potential (mV)	−2.37 ± 0.35	1.95 ± 0.27	−1.49 ± 0.25	2.76 ± 0.43
EE (%)	ND	ND	17.19 ± 1.32	19.34 ± 1.21
LC (%)	ND	ND	0.17 ± 0.03	0.08 ± 0.04
Molecular ratio of YM155 to NCs	ND	ND	1.6 ± 0.2	1.8 ± 0.5

*Note*: Data were expressed as mean ± *SD* (*n* = 3). Abbreviations: EE, encapsulation efficiency; LC, loading content; NC, nanocage; ND, not determined.

The morphology and size of all the NCs are shown in Figure 2b,c. TEM images revealed that all the NCs possessed a nanosphere‐like shape with a uniform size distribution, and DLS analysis revealed the hydrodynamic diameter of FTH1 NCs (11.2 ± 1.0 nm), FTH1/YM155 NCs (14.4 ± 1.6 nm), FTS NCs (28.1 ± 3.2 nm), and FTS/YM155 NCs (31.0 ± 3.4 nm); nanoparticles 10–100 nm in diameter are designed specifically for intravenous delivery and can be enriched in tumor tissues.^34^ In addition, zeta potential measurements were performed, and no significant difference was found among the NCs. Furthermore, the UV–Vis absorption spectrum showed that FTS/YM155 NCs had characteristic absorption peaks of YM155 (Figure 2d), indicating that YM155 was effectively encapsulated in the internal cavity of FTS NCs, and the CD spectra of FTS NCs were analogous to those of FTS/YM155 NCs (Figure 2e), suggesting that genetic fusion and YM155 encapsulation does not alter the characteristic double negative peaks of the α‐helix at 208 and 222 nm of FTS/YM155 NCs.^35^

### 3.3 NC stability and in vitro drug release

FTH1 NCs could disassemble into protein subunits under acidic conditions and release the encapsulated drugs.^36^ To observe the NC stability and the drug release, the NCs were incubated with PBS at pH 7.4 and pH 5.0, which represent the physiological condition of normal cells and lysosomes, respectively.^28^ As shown in Figure 2f, FTS/YM155 NCs and FTH1/YM155 NCs were relatively stable at pH 7.4 within 72 h and released only 14.44 ± 2.16% of YM155 from FTS/YM155 NCs. In contrast, the cumulative release rate of YM155 was markedly increased at pH 5.0 and reached a maximum release of 84.00 ± 3.80% from FTS/YM155 NCs at 72 h, which was slightly lower than that of FTH1/YM155 NCs. This might be attributed to genetic modification of TmSm on the surface of FTH1 NCs. These results indicated that FTS/YM155 NCs facilitates entry into the acidic tumor microenvironment to release the encapsulated drug but not into the neutral environment of blood. In addition, FTS/YM155 NCs and FTH1/YM155 NCs could maintain the initial particle size in PBS and 50% FBS over 48 h incubation (Figure 2g), implying that the NCs have good stability and extend the half‐life of YM155 in the blood.

### 3.4 Cleavage kinetic profile of MMP‐2 conditioned FTS NCs

**FIGURE 3:**
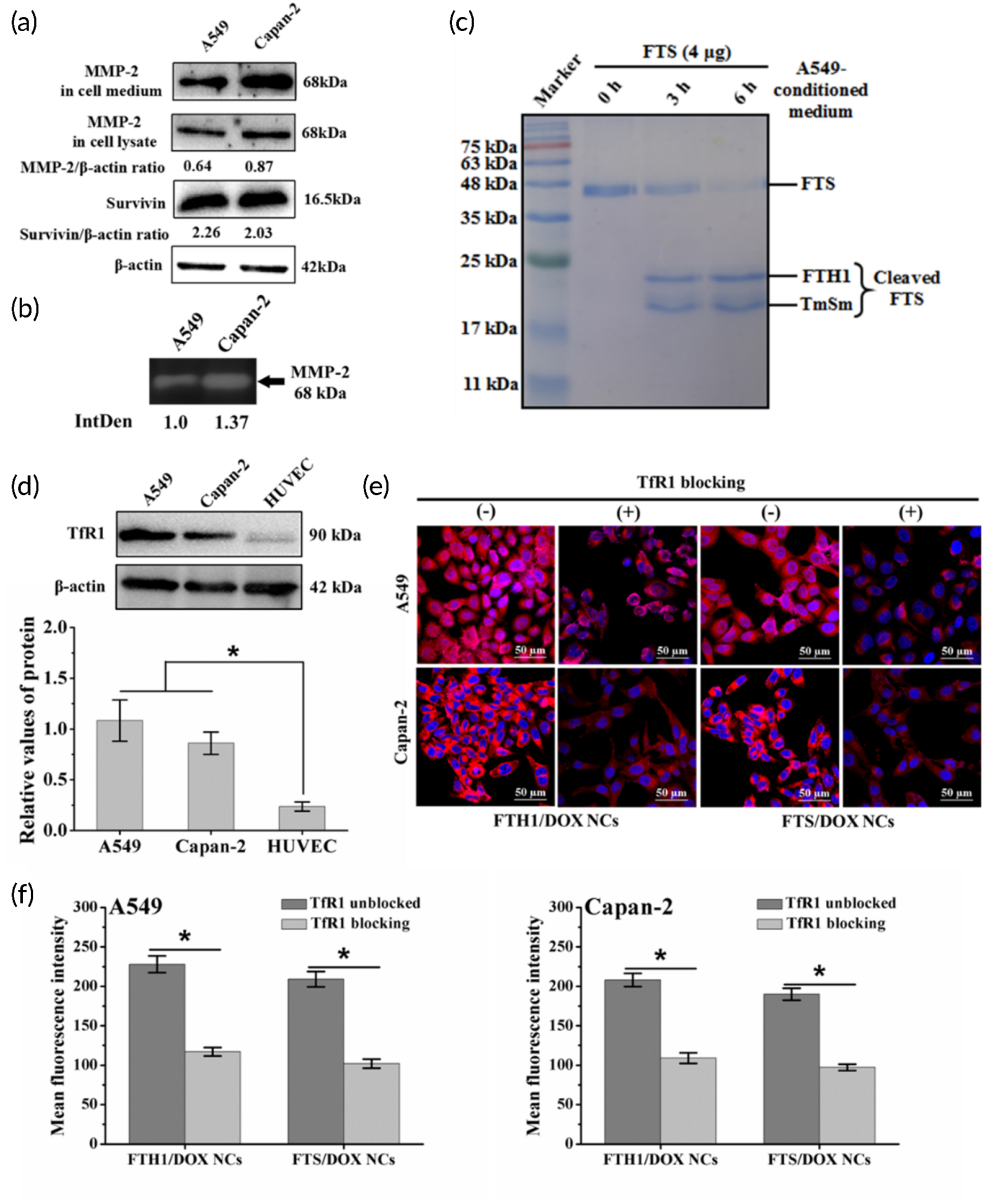
In vitro MMP‐2‐mediated cleavage and cellular uptake of FTS NCs. (a) MMP‐2 and survivin expression analyzed by Western blot. (b) Gelatin zymography analysis of secreted MMP‐2 in the conditioned medium of A549 and Capan‐2 cells. (c) MMP‐2‐catalyzed cleavage test of FTS NCs incubated with A549‐conditioned medium for 3 and 6 h, respectively. (d) TfR1 expression analyzed by Western blot. (e) Representative cellular fluorescence imaging of A549 and Capan‐2 cells incubated with FTH1/DOX NCs and FTS/DOX NCs in the absence or presence of anti‐TfR1 mAb. Red and blue colors indicate DOX and Hoechst 33342, respectively (scale bar = 50 μm). (f) The fluorescence measurements of the FTS/DOX NCs and FTH1/DOX NCs shown in (e). Data were expressed as mean ± *SD* (*n* = 3). *p < 0.05. DOX, doxorubicin; MMP‐2, matrix metalloproteinase‐2; NC, nanocage; TfR1, transferrin receptor 1

To confirm the secretion of MMP‐2 by cancer cells, MMP‐2 expression was detected. Western blot results indicated that MMP‐2 was not only expressed in Capan‐2 and A549 cells but also secreted in the culture medium (Figure 3a), as previously reported.^31^ Moreover, secreted MMP‐2 could decompose gelatin in an SDS‐PAGE gel according to gelatin zymography analysis; notably, the level of MMP‐2 secreted from Capan‐2 cells was 1.37‐fold more than that from A549 cells (Figure 3b). To further verify the cleavage of FTS NCs by secreted MMP‐2, FTS NCs were incubated with A549‐conditioned medium containing secreted MMP‐2 (Figure S6a). The results showed that FTS NCs were cleaved into FTH1 and TmSm fragments after 3 h of incubation, and more than 80% of FTS NCs were successfully cleaved (Figure 3c) after extending to 6 h. In addition, the MMP‐2‐catalyzed cleavage kinetic profile of FTS NCs was investigated in the conditioned medium of A549 cells, and the cleavage rate of FTS NCs reached 81.3% at 48 h (Figure S6b).

### 3.5 Cellular uptake of NCs mediated by TfR1 binding

To evaluate the intracellular delivery of FTH1‐based NCs, the cellular uptake of DOX‐loaded FTS and FTH1 NCs (FTS/DOX and FTH1/DOX NCs) was investigated. The fluorescence signal of FTS/DOX NCs and FTH1/DOX NCs was obviously stronger than that of free DOX and markedly increased as the treatment time increased (Figure S7a). Moreover, compared to free DOX, FTS/DOX NCs, and FTH1/DOX NCs after 12 h of treatment exhibited 4.48‐fold and 4.88‐fold higher signals for A549 and 4.36‐fold and 4.77‐fold for Capan‐2, respectively (Figure S7b). However, no significant difference was observed between NC formulations, indicating that the genetic modification of the surface of FTH1 NCs had no effect on the cellular uptake of NCs.

To verify the cellular uptake of NCs mediated by TfR1 binding, a competition binding assay was performed. As shown in Figure 3d, TfR1 expression on A549 and Capan‐2 cells was 4.5‐fold and 3.6‐fold, respectively, compared to HUVECs with low TfR1 expression. After the blockade of TfR1, the red fluorescence signal of both cells was significantly decreased (Figure 3e). Quantitative analysis showed a twofold decrease in comparison to that of the unblocked control cells (Figure 3f). As a result, dual‐targeting MMP‐2 and TfR1 improved the accuracy of tumor targeted therapy. On the one hand, MMP2 could cleave MMP‐2 sensitive peptide (G‐P‐L‐G‐L‐A‐G) and released TmSm protein. On the other hand, FTH1 NCs could specifically bind to cancer cells by interacting with TfR1. The combination of MMP2 and TRF could exert a stronger killing effect at the tumor site.

### 3.6 Anticancer effect of FTS/YM155 NCs in vitro

**FIGURE 4:**
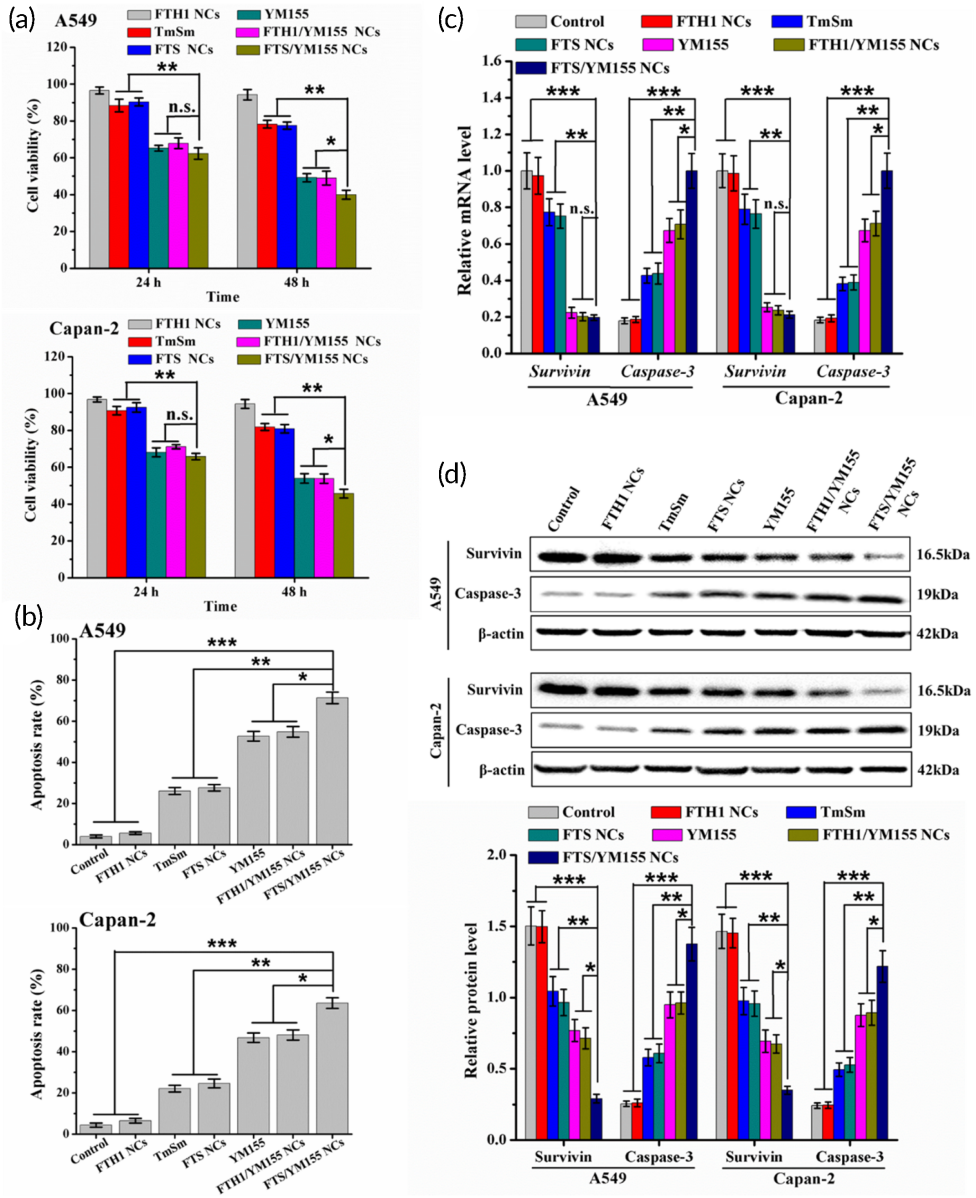
Cytotoxicity and apoptosis assay of FTS/YM155 NCs. (a) Cell viability of A549 and Capan‐2 cells incubated with TmSm, YM155, FTH1 NCs, FTS NCs, FTH1/YM155 NCs, and FTS/YM155 NCs (0.08 μM) for 24 and 48 h. The final concentrations of TmSm and YM155 were kept at 2 and 0.15 μM, respectively. (b) Apoptosis rates of A549 and Capan‐2 cells treated with TmSm, YM155, FTH1 NCs, FTS NCs, FTH1/YM155 NCs, and FTS/YM155 NCs (0.08 μM) for 48 h analyzed by Annexin V‐FITC/PI method. (c) Relative mRNA and (d) protein expression levels of survivin and caspase‐3 in A549 and Capan‐2 cells incubated with different treatments for 48 h. Data were expressed as mean ± *SD* (*n* = 3). *p < 0.05, **p < 0.01, and ***p < 0.001. NC, nanocage; n.s., not significantly different; PI, propidium iodide

To verify whether FTS NCs and FTS/YM155 NCs exerted cytotoxicity against A549 and Capan‐2 cells, an MTT assay was carried out. Both NC formulations displayed increasing cytotoxicity with increasing concentrations and exposure times (Figure S8a). Especially after 48 h of treatment, the IC_50_ values of FTS/YM155 NCs (0.063 and 0.083 μM for A549 and Capan‐2 cells, respectively) were lower than those of FTS NCs (0.253 and 0.303 μM for A549 and Capan‐2 cells, respectively) (Figure S8b). Besides, the IC_50_ value for A549 cells was lower than that for Capan‐2 cells. It might be due to the differential expression of survivin in Capan‐2 cells (2.03‐fold) and A549 cells (2.26‐fold) (Figure 3a). Furthermore, FTS/YM155 NCs showed higher cytotoxicity than FTH1 NCs and FTH1/YM155 NCs (*p* < 0.05) and TmSm protein and FTS NCs (*p* < 0.01), which could be attributed to the synergistic effect of TmSm protein and YM155 (Figure 4a).

The apoptotic effect of FTS/YM155 NCs in A549 and Capan‐2 cells was conducted by an Annexin V‐FITC/PI double staining assay. The percentage of apoptotic cells was significantly increased with FTS/YM155 NCs treatment versus FTH1 NCs and FTH1/YM155 NCs treatment (*p* < 0.05) and TmSm protein and FTS NCs treatment (*p* < 0.01) (Figure S9a,b). Moreover, FTH1 NCs only induced a low level of apoptosis in both cells, indicating that it was a safe intravenous delivery system. Moreover, the apoptosis rate of A549 cells treated with FTS/YM155 NCs was increased by 7.77% compared to that of Capan‐2 cells (Figure 4b), which was consistent with the MTT results. The mRNA and protein expression levels of survivin and caspase‐3 in cancer cells were analyzed using qRT‐PCR and Western blot. The data showed that YM155, FTH1/YM155 NCs, and FTS/YM155 NCs significantly reduced survivin mRNA expression and also decreased survivin protein expression (Figure 4c,d). Notably, FTS/YM155 NCs treatment had the most obvious inhibitory effect on survivin expression at both mRNA and protein levels and could distinctly upregulate caspase‐3 expression. Taken together, these findings indicated that FTS/YM155 NC treatment had greater benefits than free drug and single‐drug‐loaded NC treatment.

### 3.7 In vivo distribution and therapeutic efficacy of NCs

**FIGURE 5:**
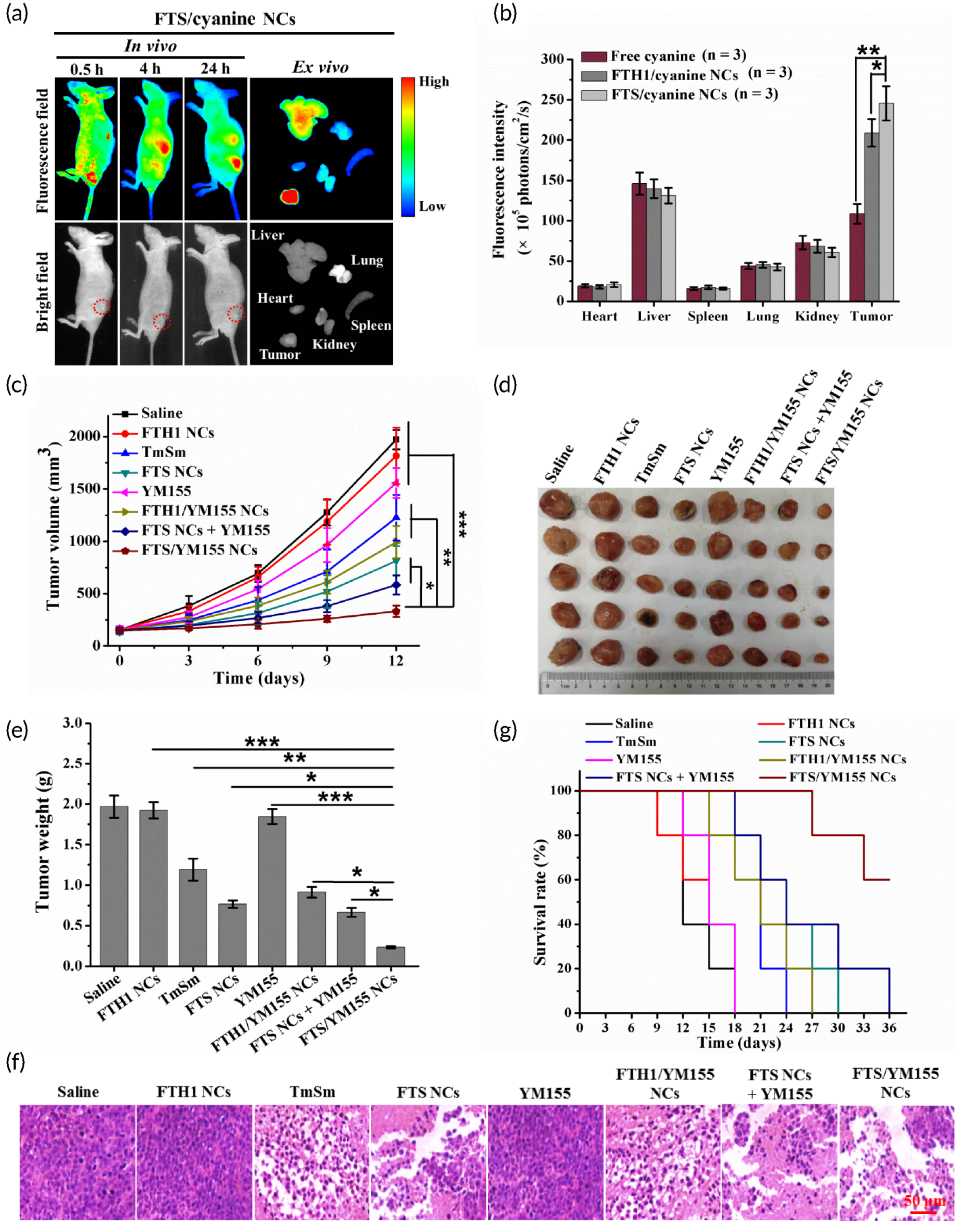
In vivo imaging for tumor targeting ability and antitumor efficacy of FTS/YM155 NCs. (a) In vivo imaging of A549 tumor‐bearing mice after intravenous injection of FTS/cyanine NCs (cyanine, ex/em = 740/830 nm) at 0.5, 4, and 24 h, respectively, and ex vivo imaging of dissected organs and tumors at 24 h postinjection. Red circle marks the tumor location. (b) Quantitative analysis of fluorescence intensity of NCs in major organs and tumors. (c) Changes in tumor volume treated with different drugs. (d) Photographs and (e) weight of tumors collected from A549 tumor‐bearing mice in each group (Day 12). (f) Representative HE staining of tumor tissue sections in different treatments (Day 12). All images are taken at ×400 magnification (scale bar = 50 μm). (g) The survival rate of mice treated with different drugs. Data were expressed as mean ± *SD* (*n* = 5). *p < 0.05, **p < 0.01, and ***p < 0.001. HE, hematoxylin and eosin; NC, nanocage

To evaluate tissue uptake, the biodistribution of cyanine‐loaded NCs in A549 tumor‐bearing mice was determined. The fluorescent signal in the tumor sites gradually increased with time after tail vein injection of free cyanine and cyanine‐loaded NCs, and higher levels of fluorescence were observed in the tumors at 24 h after injection (Figures 5a and S10a). In particular, the liver exhibited high uptake, as indicated by a high fluorescence intensity, which was in accordance with previous reports.^36^ Moreover, the fluorescence intensity of tumors treated with the FTS/cyanine NCs was significantly higher than that of those treated with FTH1/cyanine NCs and free cyanine (Figure 5b), suggesting that FTS/cyanine NCs had a better tumor‐targeting effect. The accumulation of FTS/cyanine NCs in tumor tissues could be attributed to their smaller size (31.0 ± 3.4 nm), which enables their internalization to the tumor sites via the enhanced permeability and retention (EPR) effect, and interference with the intrinsic ability of FTS NCs to gravitate toward physiological cells overexpressing TfR1, such as activated lymphocytes and erythroid precursors, as also observed in previous studies.^34^, ^37^

Based on better anticancer effect in vitro, we were trying to reflect the in vivo therapeutic potential of these NCs in clinical settings. As shown in Figure 5c, tumor growth was obviously inhibited in the FTS/YM155 NC treatment group compared to the other treatment groups. The average tumor size at Day 12 in the FTS/YM155 NC group was 331.7 ± 54.0 mm^3^, while it was 583.7 ± 91.6, 992.7 ± 154.5, 814.8 ± 141.0, and 1226.6 ± 216.5 mm^3^ in the FTS NC + YM155, FTH1/YM155 NC, FTS NC, and TmSm treatment groups, respectively. FTS/YM155 NCs had the highest tumor growth inhibition rate (88.86 ± 5.94%, Figure 5d,e and Table S2). Moreover, FTS/YM155 NC‐treated mice showed the most obvious cell necrosis and largest interstitial space (Figure 5f). Importantly, compared with other treatments, FTS/YM155 NCs prolonged the survival time of mice (Figure 5g). Also, there was no significant body weight loss in these several NCs‐treated mice (Figure S10b). These results highlighted the effect of the codelivery of TmSm and YM155, which was superior to that of individual drugs and single‐drug delivery systems.

### 3.8 Mechanism underlying the efficacy of FTS NC‐based therapy

**FIGURE 6:**
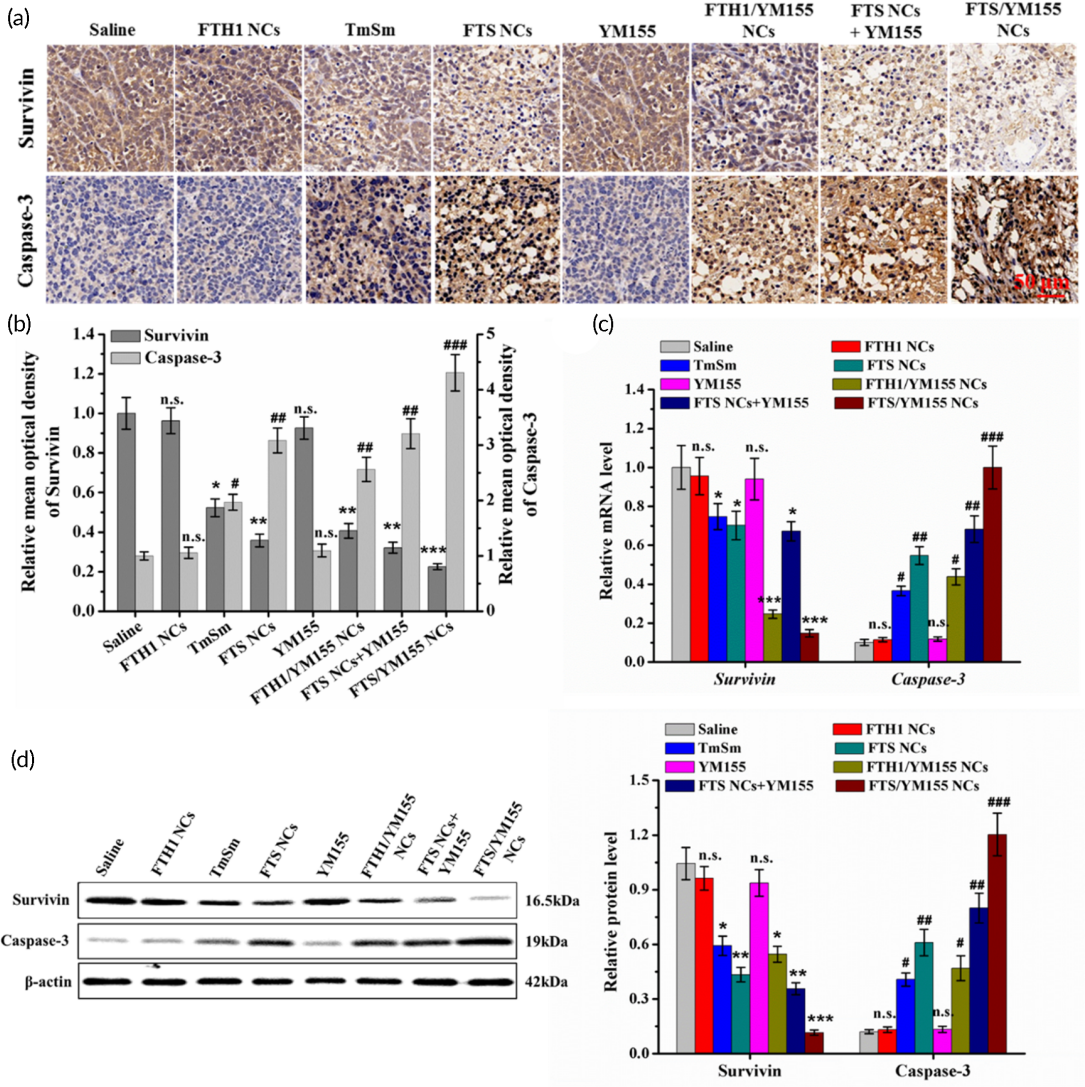
Mechanism of the treatments underlying the efficacy of FTS NCs‐based therapy. (a) Representative survivin and caspase‐3 expression of tumor tissue sections treated with different drugs. All images are taken at ×400 magnification (scale bar = 50 μm). (b) Immunohistochemical index quantitative analysis of caspase‐3 and survivin expression of tumor tissues shown in (a) using ImageJ. (c) qRT‐PCR analysis of relative mRNA expression of caspase‐3 and survivin in tumor tissues shown in (a). (d) Western blot of survivin and caspase‐3 protein level (left) in tumor tissues shown in (a) and the corresponding quantitative analysis (right). Data were expressed as mean ± *SD* (*n* = 5). *p < 0.05, **p < 0.01, and ***p < 0.001 compared to saline group. *p < 0.05, **p < 0.01, and ***p < 0.001 compared to saline group. NC, nanocage; n.s., not significantly different; qRT‐PCR, quantitative real‐time polymerase chain reaction

We further explored the potential mechanism underlying the efficacy of FTS NC‐based therapy. Survivin protein expression was reduced in the TmSm, FTS NC, FTH1/YM155 NC, and FTS/YM155 NC treatment groups, while caspase‐3 expression was increased in these groups compared to the saline‐treated group (Figure 6a). Moreover, compared to saline treatment, FTS/YM155 NC treatment significantly downregulated survivin (4.43‐fold) and upregulated caspase‐3 (4.31‐fold) (Figure 6b). The qRT‐PCR and Western blot results showed that compared to FTH1/YM155 NC or FTS NC treatment, FTS/YM155 NC treatment markedly reduced survivin expression and increased caspase‐3 expression at the transcript and protein levels (Figure 6c,d). Taken together, at the transcription level, YM155 could inhibit the expression of *BIRC5* (encoding survivin protein) and reduce the production of survivin mRNA; at the protein level, TmSm protein was delivered to the cytoplasm through TAT peptide and formed a heterodimer with wild‐type survivin, competitively inhibits the formation of survivin homodimer, thereby promoting the expression of caspase‐3, and ultimately leading to cancer cell apoptosis. These results suggested that the co‐delivery of YM155 and TmSm enhanced caspase‐3 expression by synergistically inhibiting the transcript and protein expression levels of survivin, highlight the obvious in vivo antitumor activity of this approach. Biosafety is the basic evaluation requirement for drug development.^38^ Based on the hemolytic analysis, the HRs were drug concentration dependent. However, the HRs of all drugs at the highest concentration (0.33 μM) were less than 5% (Figure S11a,b). In addition, there was no apparent histopathological changes (Figure S12) in the main organs in any of the groups. These results suggested that FTS/YM155 NCs inhibit tumor growth without inducing major organ injury.

## 4 DISCUSSION

Survivin has been regarded as an ideal target for tumor therapy due to its overexpression in most human cancers but not in most normal tissues; it can inhibit the activation of caspase‐7 and caspase‐3 by binding to caspase‐9 to inhibit the apoptosis process.^39^ Most survivin‐targeting approaches have been aimed at reducing survivin expression using siRNA and other forms of gene therapy.^6^ However, the clinical antitumor efficacy of such monotherapies has been limited. There is an obvious evidence that combination strategies with chemotherapy or other targeted therapies can enhance the antiproliferative effect, especially by circumventing drug resistance.^15^, ^40^ In the present study, we developed an FTH1 protein‐based system enabling co‐delivery of the TmSm protein and YM155 to obviously inhibit the transcript and protein levels of survivin in A549 and Capan‐2 cells. On the one hand, as a novel type of survivin inhibitor, YM155 can inhibit the expression of survivin at the transcription level, thereby reducing its protein expression level.^11^ On the other hand, TmSm is the most effective survivin mutant, which can form heterodimerization with wild‐type survivin protein, thereby decreasing the formation of homodimers of survivin protein. Both YM155 and TmSm ultimately affect the formation of survivin homodimers.^12^, ^13^ FTH1 NCs are amenable to reconstitution through pH‐dependent assembly/disassembly, which circumvents the need for dissolution in organic solvents, sonication, or stirring, which are used in the preparation of other nanoparticle types.^41^, ^42^ Moreover, FTH1 NCs carrying the TmSm protein can be easily scaled up by *E. coli* expression and subsequent purification. CD spectra, TEM images, and in vitro drug release tests demonstrated that the genetic functionalization of the TmSm protein did not affect the intrinsic properties of FTH1 NCs, including the NC structure and assembly/disassembly.^19^ Importantly, the genetically functionalized FTH1 NCs showed a higher EE of YM155 than the FTH1 NCs, which might be related to the blockage of hydrophilic and hydrophobic channels on the surface of FTH1 NCs by the TmSm protein, which prevents the leakage of small molecule drugs.^43^ Similarly, FTH1 NCs have been demonstrated to be promising anticancer drug delivery platforms due to their natural TfR1 targeting without any ligand functionalization.^21^ However, other NCs are usually modified with targeting ligands to specifically bind to overexpressed receptors, such as folate receptor, integrin, human epidermal growth factor receptor 2, and TfR1, on the surface of tumor cells.^44^ In our studies, FTH1 NCs loaded with DOX could effectively accumulate in the nucleus of cancer cells, in accordance with a previous report.^24^ However, systemic delivery of FTH1‐based NCs might cause adverse effects due to high expression of TfR1 on many physiological cells in the blood circulation, including activated lymphocytes and erythroid precursors.^37^ Moreover, the indiscriminate penetration of TmSm protein could cause damage to several types of physiological cells overexpressing survivin, such as primitive hematopoietic cells, vascular endothelial cells, and T lymphocytes.^10^, ^30^ In our design, an MMP‐2‐sensitive peptide was introduced between the TmSm protein and FTH1 protein to effectively overcome the adverse side effects of TmSm and FTH1 NCs. Thus, the TmSm protein could be released in the tumor microenvironment through the MMP‐2 enzyme response and could be released into the cytoplasm through the TAT peptide.^30^

The in vivo behavior of NCs is largely determined by their size and surface charge. It has been reported that NCs with a weak positive charge and <100 nm diameter can optimally exploit the EPR effect.^34^, ^45^ According to reports, silica NCs with a diameter of 50 nm have the highest tumor penetration and tumor cell uptake and the slowest tumor clearance among three different sizes of NCs (20, 50, and 200 nm).^46^ Compared to these constructs, our prepared FTS/YM155 NCs had a small NC structure (31.0 ± 3.4 nm) and had better cellular uptake, longer circulating half‐life, and higher tumor accumulation rate in different types of tumor tissues, especially pancreatic adenocarcinoma tissues, during intravenous administration.^47^ In vivo imaging demonstrated that FTS/YM155 NCs could effectively accumulate in the tumor tissues of A549 tumor‐bearing mice, thereby significantly inhibit tumor growth. Meanwhile, As reported in previous studies, Su et al.^48^ found that the calculated half‐lives of FITC‐labeled FTH1 and GLP‐FTH1 were 51.1 and 51.9 h, respectively. The long half‐lives may be the result of slow absorbance by the capillaries after subcutaneous injection due to the large hydrodynamic diameter. Wang and his colleagues showed that FTH1/DOX had better pharmacokinetic behavior than free DOX.^43^ Also, FTH1‐DOX had been shown to have a longer plasma half‐life and higher area under the concentration time curve, which might improve the retention of the drug in the systemic circulation and promote the time‐dependent accumulation of the drug in the tumor.^21^ Taken together, FTH1 NCs could increase the pharmacokinetic behavior of free drugs.

We further sought to determine whether simultaneous delivery of TmSm protein and YM155 by FTS NCs could synergistically decrease survivin expression at both transcript and protein levels. We found that the cell viability of A549 cells treated with TmSm and YM155 alone for 48 h was 78.3% and 49.2%, respectively, while the cell viability of FTS/YM155 NCs was 40%. These result above implied that TmSm and YM155 had a synergistic effect. Because if the functions of TmSm and YM155 are additive, the viability of A549 cells treated with FTS/YM155 NCs should be about 27.5%, actually 40%. However, in the apoptosis experiment, we found that the apoptosis rate of A549 cells treated with TmSm and YM155 alone for was 26% and 52.6%, respectively, and the apoptosis rate of FTS/YM155 NCs was 71.3%. Since the treatment of TmSm, YM155, and FTS/YM155 all ultimately led to a decrease in the level of survivin protein, which in turn led to cell apoptosis, so the level of apoptosis was partially additive. As a result, FTS/YM155 NCs could effectively inhibit the transcription and protein level of survivin, indicating a synergistic effect, but the final downregulation of survivin could also be said to have an additive effect. Meanwhile, several results indicated that compared to single‐drug delivery, the co‐delivery of TmSm protein and YM155 significantly inhibited survivin expression and further enhanced caspase‐3 in the tumor tissues. As a member of the IAP family, the TmSm protein can dissociate the caspase‐9–survivin complex by removing the phosphorylation of endogenous survivin on threonine‐34 and induce caspase‐dependent apoptosis.^49^ YM155, a well‐known inhibitor of survivin, is reported to repress survivin expression at the transcriptional level by preventing the binding of the transcription factor Sp1 to the core promoter region located 71–149 bp upstream of the transcription initiation site (‐149‐71).^11^ In vivo studies demonstrated that YM155 and TmSm protein co‐delivered by FTH1 NCs had more obvious anticancer effects than single‐loaded NCs (FTS NCs and FTH1/YM155 NCs) and free drugs (TmSm protein and YM155). As a result, compared with other studies of FTH1 loaded drugs, FTS/YM155 has stronger cell killing ability. For example, the FTH1‐PTX NPs could inhibit the growth of MDA‐MB‐231 cancer cells and the IC_50_ value was 1.67 ± 0.10 μg/ml for 48 h.^50^ The IC_50_ of 3LL cells treated with ferritin heavy chain nanocages (FTn)/FTn‐PEG2k/DOX for 24 h was 1.1 μM.^37^ However, in the present study, the IC_50_ of A549 cells treated with FTS/YM155 for 24 and 48 h were 0.154 and 0.063 μmol/L, respectively (Figure S8b). This means that FTS/YM155 NCs with dual inhibition of survivin has a stronger antitumor effect. The fact that tumor volume in the FTS/YM155 NC group showed a greater decrease than that in the other groups suggested that the FTH1 NC delivery platform greatly enhanced the antitumor effect. Downregulated survivin expression at both mRNA and protein levels in the tumors was found to reduce tumor growth. In our previous study, the TmSm protein acted as a chemosensitizer to significantly enhance the cytotoxicity of DOX to breast cancer cells.^51^ Several other groups have also reported that survivin inhibition can reverse the resistance of cancer cells against chemotherapeutic drugs.^52^, ^53^ These results and ours indicate that clinical first‐line chemotherapeutic drugs, such as DOX, paclitaxel, and gemcitabine, can also be delivered by FTS NCs for tumor‐targeted therapy. This approach may also prevent possible side effects caused by high‐dose drug treatment.

## 5 CONCLUSION

In summary, we successfully developed MMP‐2‐sensitive ferritin heavy chain nanocages (FTS/YM155 NCs) by fusing TmSm to the C‐terminus of the *FTH1* gene via an MMP‐2‐sensitive peptide and encapsulating YM155 in hollow FTH1 NCs; this method can easily be scaled up to the clinical application level. FTS/YM155 NCs were cleaved in the tumor sites by secreted MMP‐2 to release TmSm and actively targeted cancer cells via TfR1 binding. Tumor targeting enhanced FTS/YM155 NC accumulation in cancer cells and decreased accumulation in major organs in A549 tumor‐bearing mice. In addition, compared with YM155 or TmSm alone, FTS/YM155 NCs significantly improved the anticancer effect by synergistically downregulating survivin at the transcript and protein levels in vitro and in vivo. This therapeutic strategy uses a tumor microenvironment‐responsive platform for multilevel inhibition of cancer targets; it shows great promise for cancer treatment and can be used in the future to target other biomarkers.

## AUTHOR CONTRIBUTIONS

**Fabiao Hu:** Data curation (lead); formal analysis (lead); investigation (lead); methodology (lead); software (lead); validation (lead); writing – original draft (lead); writing – review and editing (lead). **Changping Deng:** Data curation (supporting); investigation (supporting); methodology (supporting); supervision (supporting); writing – original draft (equal); writing – review and editing (lead). **Yiwen Zhou:** Data curation (supporting); formal analysis (supporting); investigation (supporting); software (supporting); writing – original draft (supporting). **Yuping Liu:** Data curation (supporting); investigation (supporting); software (supporting). **Tong Zhang:** Data curation (supporting); investigation (supporting); supervision (supporting). **Peiwen Zhang:** Data curation (supporting); investigation (supporting); visualization (supporting). **Zhangting Zhao:** Data curation (supporting); investigation (supporting); methodology (supporting); supervision (supporting). **Hui Miao:** Formal analysis (supporting); software (supporting). **Wenyun Zheng** **:** Writing – original draft (equal); writing – review and editing (lead). **Wenliang Zhang:** Data curation (supporting); investigation (supporting); writing – review and editing (supporting). **Meiyan Wang** **:** Writing – review & editing. **Xingyuan Ma:** Conceptualization (lead); funding acquisition (lead); project administration (lead); writing – original draft (lead); writing – review and editing (lead).

## CONFLICT OF INTERESTS

The authors declare no conflict of interests.

### PEER REVIEW

The peer review history for this article is available at https://publons.com/publon/10.1002/btm2.10290.

## Supporting information


**Appendix** **S1:** Supporting Information

## Data Availability

All data needed to evaluate the conclusions in the paper are present in the paper and/or the Supplementary Materials. Additional data related to this paper may be requested from the authors.
