# Supplementary material for: Multistage targeting and dual inhibiting strategies based on bioengineered tumor matrix microenvironment‐mediated protein nanocages for enhancing cancer biotherapy
Source: Bioeng Transl Med. 2022 Jan 5;7(2):e10290. doi: 10.1002/btm2.10290 (PMC9115700; doi:10.1002/btm2.10290)
Supplement: Supplementary file 1 — Appendix S1: Supporting Information [file BTM2-7-e10290-s001.pdf]

Supplementary Information

Multistage targeting and dual inhibiting strategies based on bioengineered tumor matrix microenvironment-mediated protein nanocages for enhancing cancer biotherapy

Fabiao Hu[1*], Changping Deng[1*], Yiwen Zhou[1*], Yuping Liu[2], Tong Zhang[2], Peiwen Zhang[2], Zhangting Zhao[1], Hui Miao[1], Wenyun Zheng[2†], Wenliang Zhang[3], Meiyan Wang[4†], Xingyuan Ma[1†]

[1]*State Key Laboratory of Bioreactor Engineering, East China University of Science and Technology, Shanghai 200237, P. R. China*

[2]*Shanghai Key Laboratory of New Drug Design, School of Pharmacy, East China University of Science and Technology, Shanghai 200237, P. R. China*

[3]*Department of Genetics, Faculty of Life Science, KIM IL SUNG University, Pyongyang 999093, Democratic People's Republic of Korea*

[4]*Center of Translational Biomedical Research, University of North Carolina at Greensboro, Greensboro, North Carolina 27310, United States*

[5]*Synthetic Biology and Biomedical Engineering Laboratory, Biomedical Synthetic Biology Research Center, Shanghai Key Laboratory of Regulatory Biology, Institute of Biomedical Sciences and School of Life Sciences, East China Normal University, Shanghai 200241, P. R. China*

[6]*Department of Chemical & Biomolecular Engineering, National University of Singapore, 117585, Singapore*

* These authors contributed equally to this work.

***Corresponding author.Email:** maxy@ecust.edu.cn(Xingyuan Ma); mywang@bio.ecnu.edu.cn(Meiyan Wang); zwy@ecust.edu.edu.cn(Wenyun Zheng)

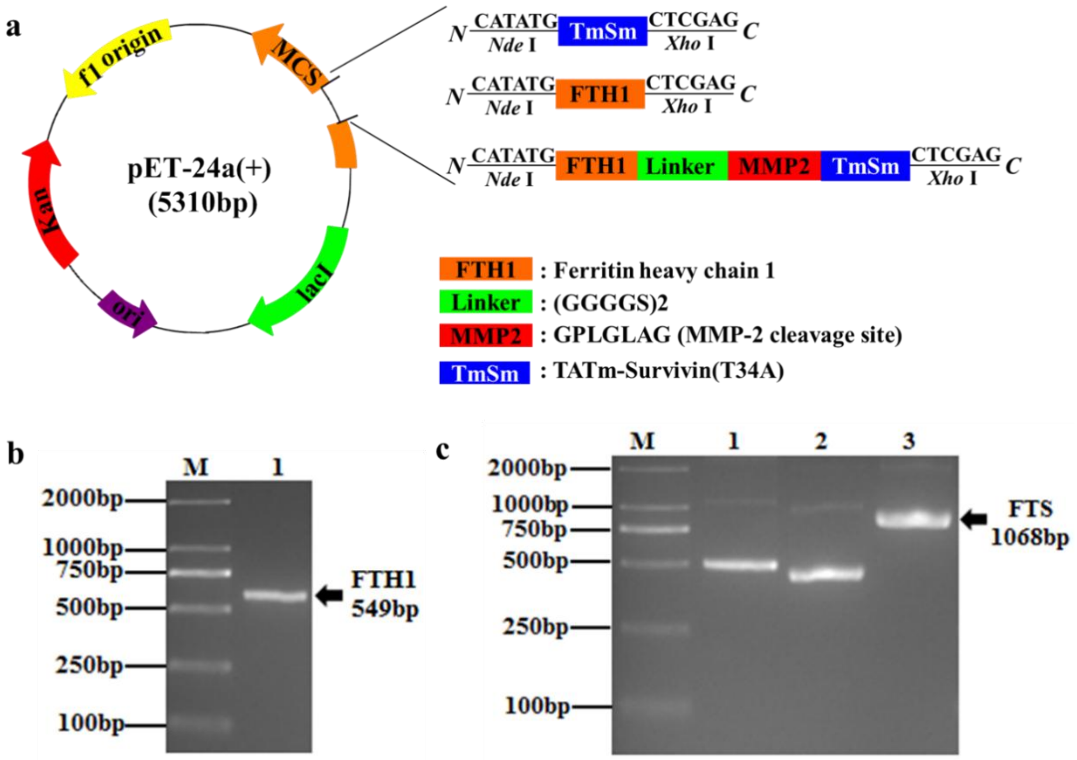

Figure S1. Construction of recombinant plasmid pET-24a(+)-FTH1 and pET-24a(+)-FTS. (a) Schematic diagram of constructing the recombinant plasmid. (b) Agarose gel electrophoresis of FTH1 *via* PCR amplification. Lane M, DL2000 marker; lane 1, FTH1 amplification fragment. (c) Agarose gel electrophoresis of FTS *via* overlap extension PCR amplification. Lane M, DL2000 marker; lane 1, FTH1-Linker amplification fragment; lane 2, MMP2-TmSm amplification fragment; lane 3, FTS amplification fragment.

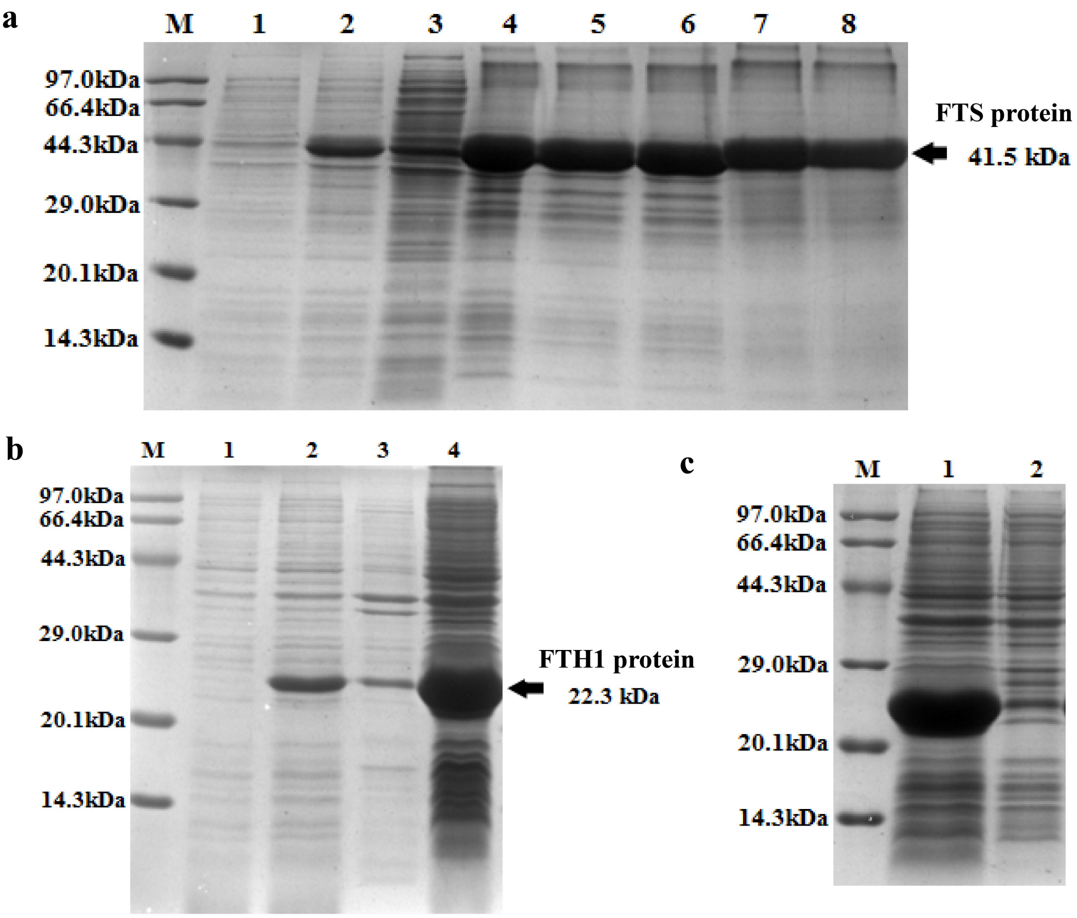

Figure S2. Expression of FTS and FTH1 proteins. (a) Expression of the recombinant FTS protein analyzed by SDS-PAGE electrophoresis. Lane M, protein size marker; lane 1, preinduction bacteria; lane 2, postinduction bacteria; lane 3, the supernatant of cell lysates; lane 4, the precipitation of cell lysates; lane 5-7, inclusion body of FTS protein after 1, 2, and 3 washings, respectively; lane 8, the dissolved inclusion body. (b) Expression of the recombinant FTH1 protein analyzed by SDS-PAGE electrophoresis. Lane M, protein size marker; lane 1, preinduction bacteria; lane 2, postinduction bacteria; lane 3, the precipitation of cell lysates; lane 4, the supernatant of cell lysates. (c) The disrupted supernatant after heat shock at 60 °C analyzed by SDS-PAGE electrophoresis. Lane M, protein size marker; lanes 1, the supernatant after hot shock; lanes 2, the precipitation after hot shock.

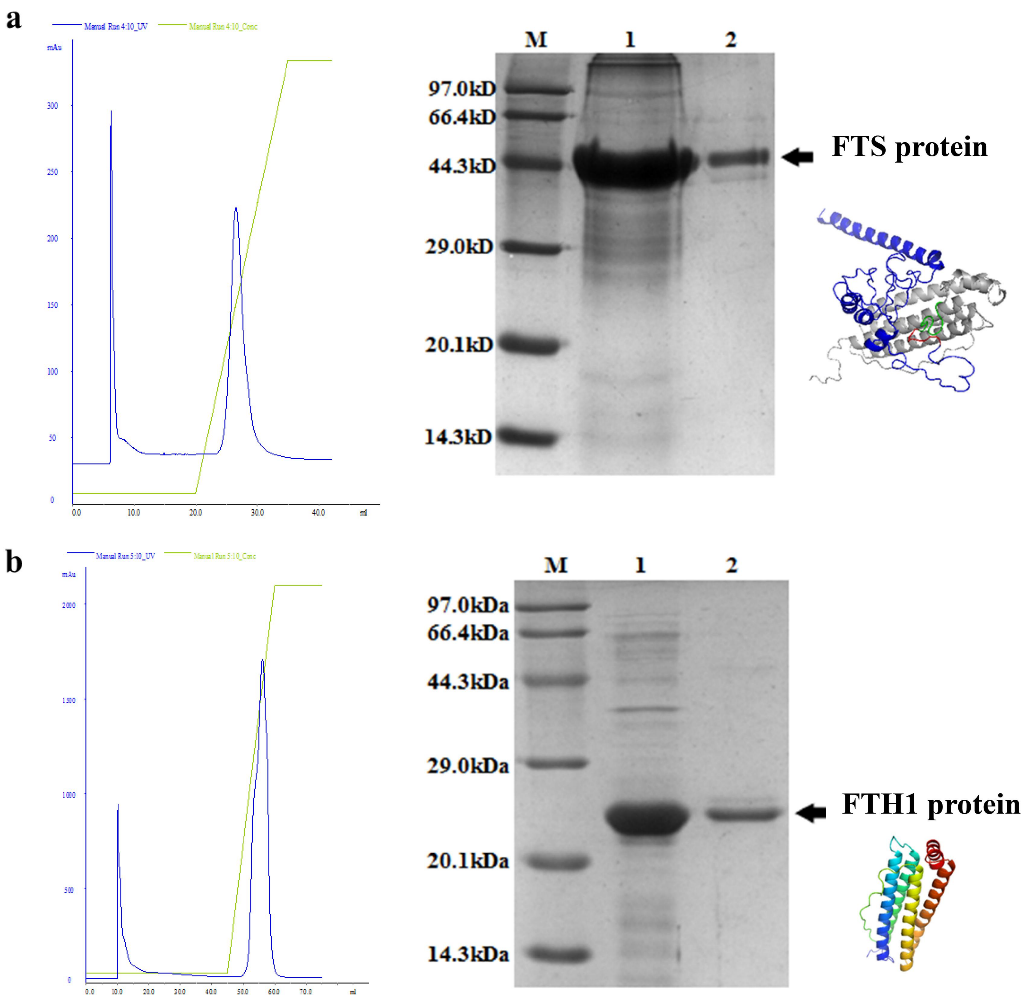

Figure S3. Purification of FTS and FTH1 proteins. (a) Purification of FTS protein by DEAE sepharose chromatogram and analysis of SDS-PAGE analysis. Lane M, protein size marker; lane 1, the dissolved inclusion body; lane 2, DEAE column eluate. (b) Purification of FTH1 protein by DEAE sepharose chromatogram and analysis of SDS-PAGE analysis. Lane M, protein size marker; lane 1, the supernatant after hot shock; lane 2, DEAE column eluate.

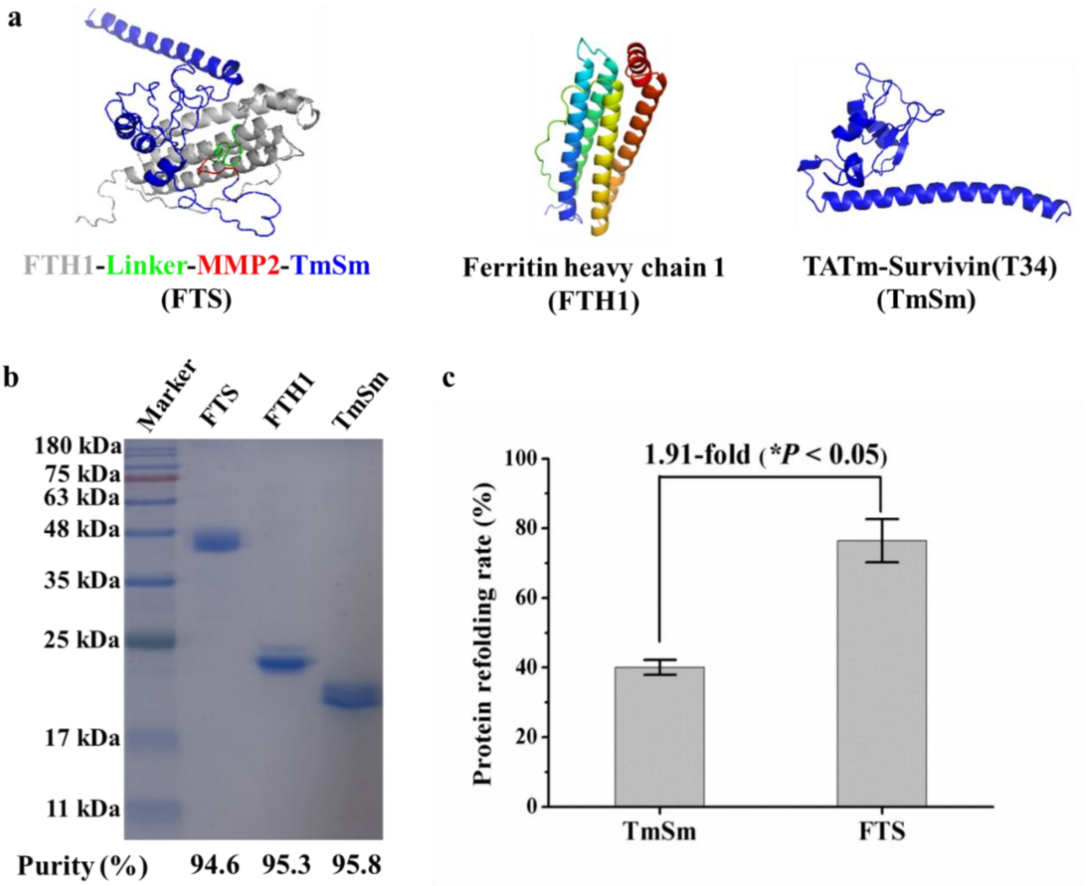

Figure S4. Three-dimensional structure simulation and preparation of the recombinant FTS, FTH1, and TmSm proteins. (a) Schematic diagram of three-dimensional structure of the recombinant FTS, FTH1, and TmSm proteins. (b) SDS-PAGE analysis of the purified FTS, FTH1, and TmSm proteins. (c) Comparison of refolding rates of FTS and TmSm proteins. Data were expressed as means ± SD (n = 3).

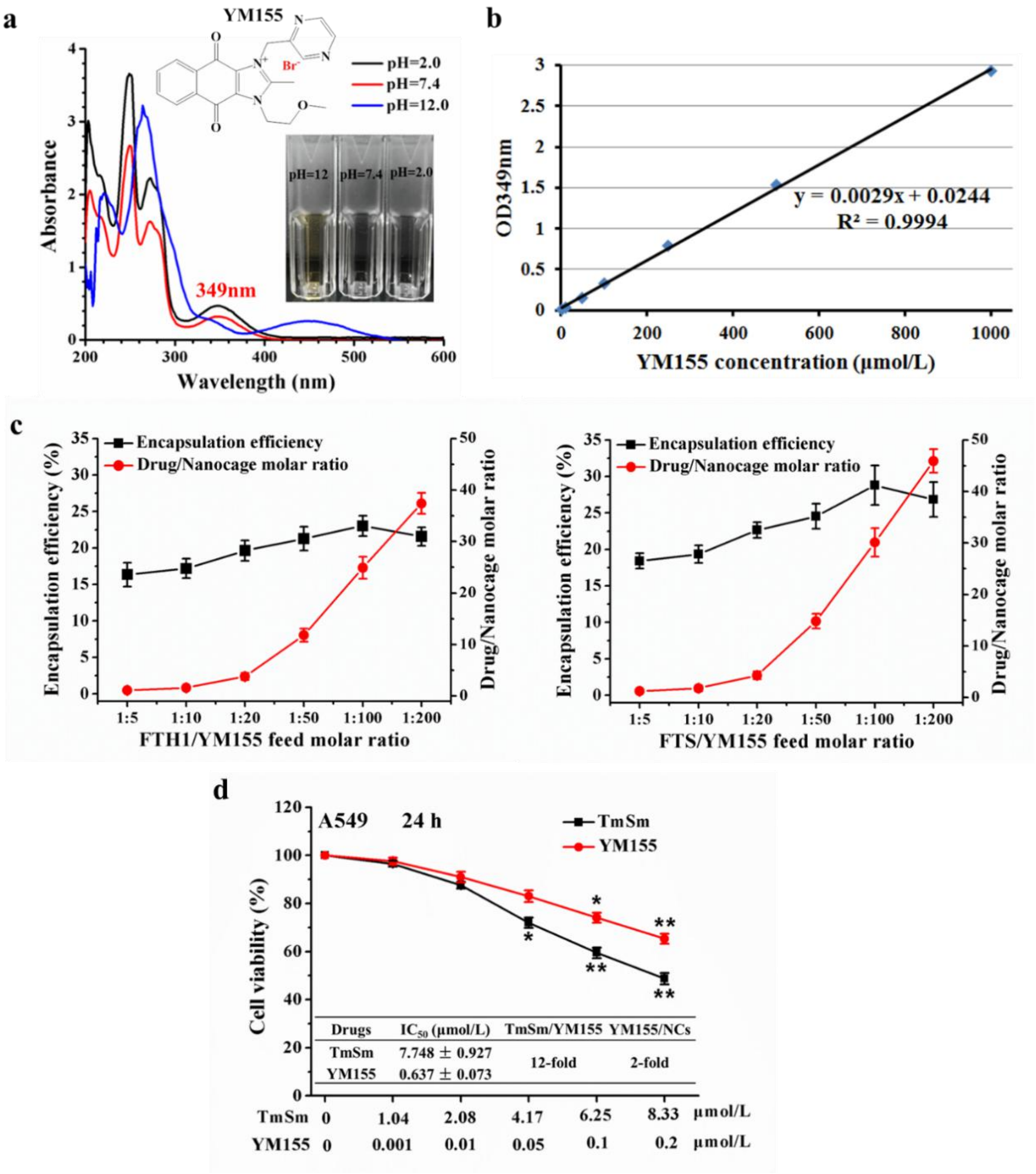

Figure S5. Preparation of FTH1/YM155 NCs and FTS/YM155 NCs. (a) Full-wavelength scanning of YM155 in different pH (pH = 2.0, 7.4, and 12.0) buffers. (b) Standard curve of YM155. (c) Encapsulation efficiency (EE) and drug/nanocage molar ratio of YM155-loaded NCs prepared at different protein/YM155 feed molar ratios. (d) Cytotoxicity assay of TmSm and YM155 against A549 cells. Data were expressed as means ± SD (n = 3). **P* < 0.05 and ***P* < 0.01.

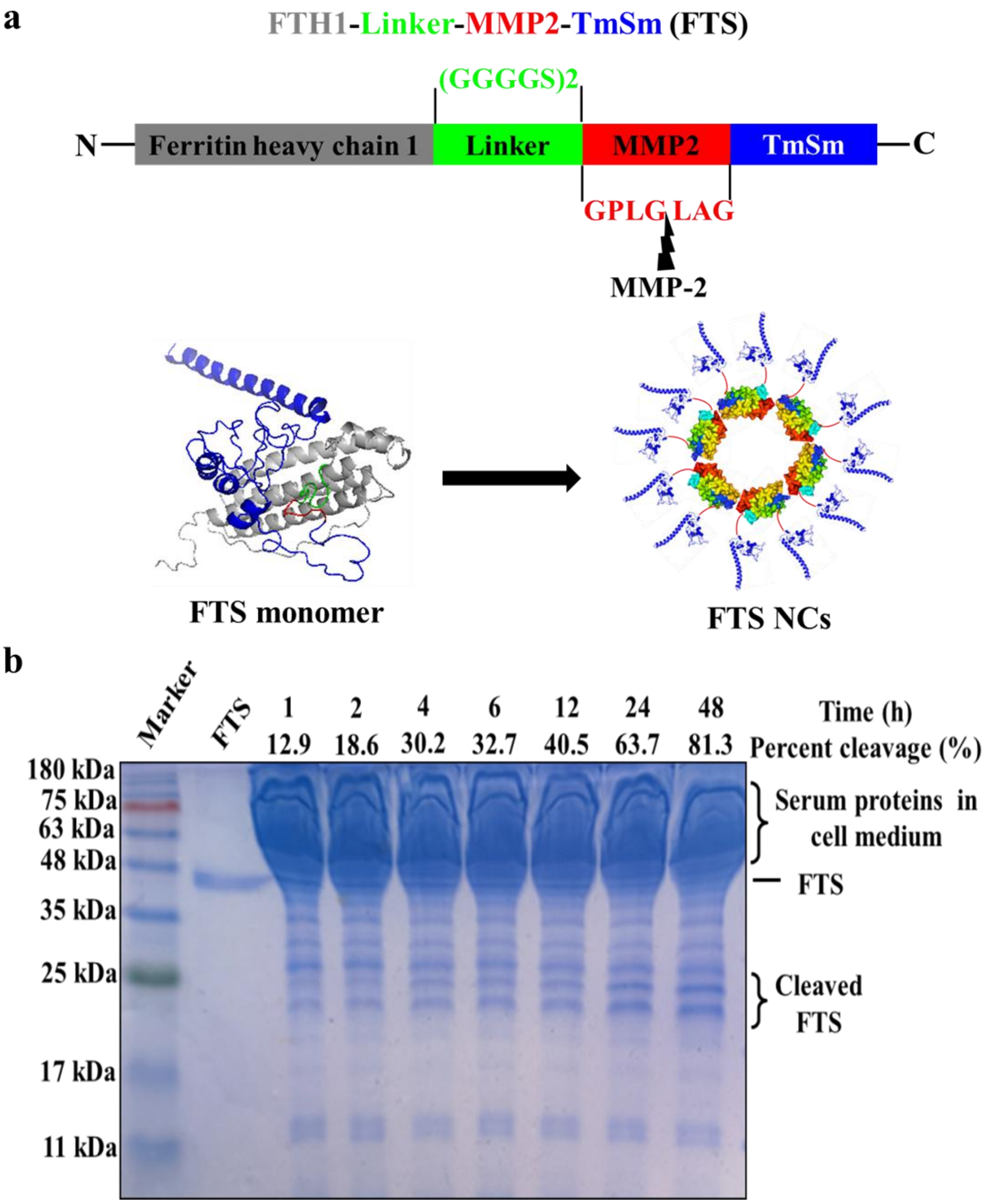

Figure S6. Cleavage of FTS NCs catalyzed by MMP-2. (a) Schematic depicting the configuration of FTS protein and MMP-2 mediated cleavage site. (b) Kinetic profile of FTS NCs cleavage catalyzed by MMP-2 using SDS-PAGE.

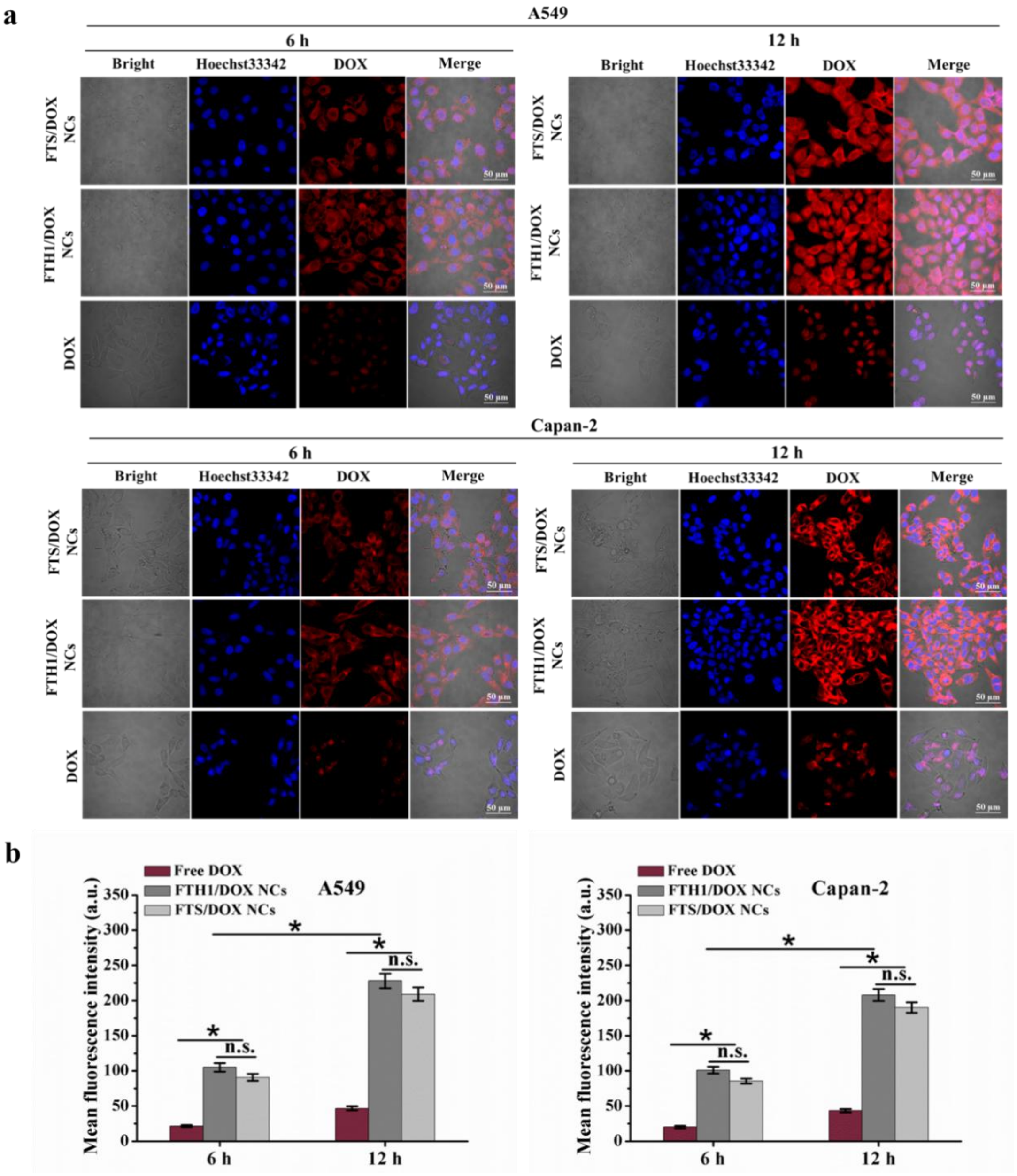

Figure S7. Cellular uptake of FTS/DOX NCs and FTH1/DOX NCs in A549 and Capan-2 cells. (a) Cellular distribution of NCs observed by CLSM. Red and blue colors indicated DOX and Hoechst33342, respectively (Scale bar = 50 μm). (b) Mean fluorescence intensity of NCs shown in A measured by flow cytometry. Data were expressed as means ± SD (n = 3). **P* < 0.05 and n.s., not significantly different.

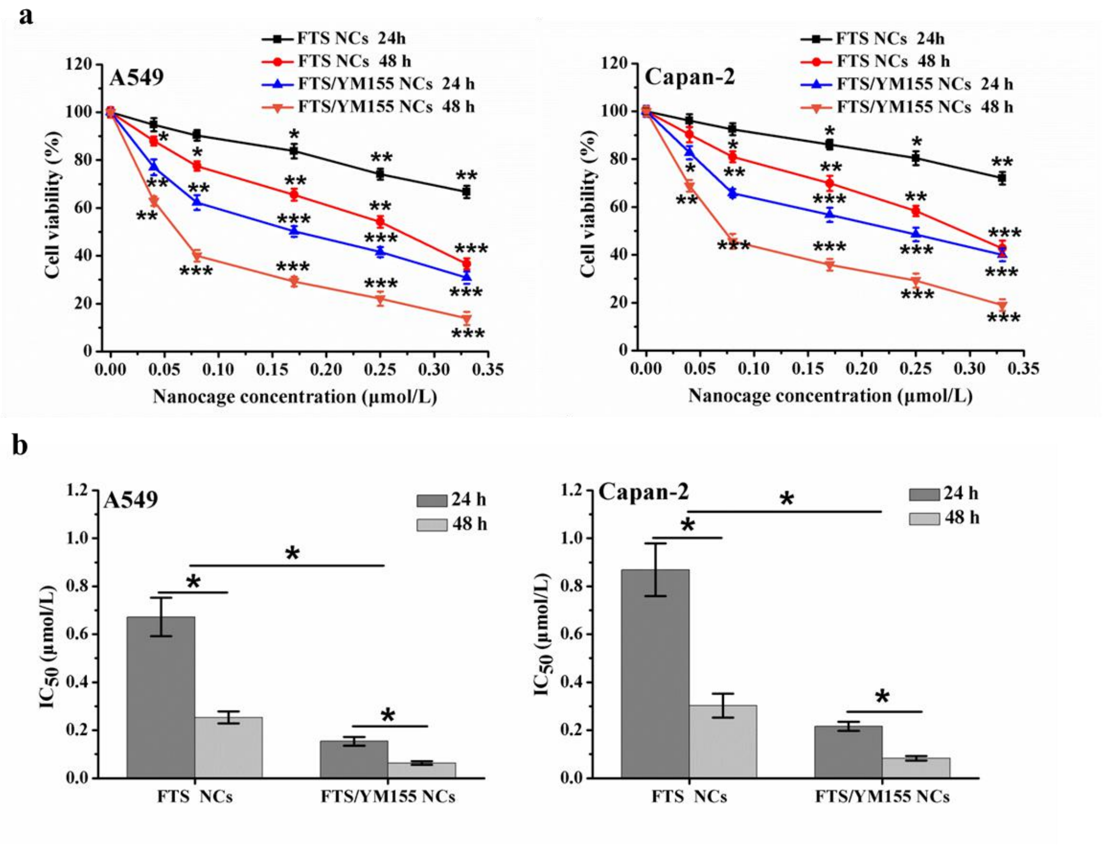

Figure S8. Cytotoxicity of FTS NCs and FTS/YM155 NCs against A549 and Capan-2 cells. (a) Cell viability of A549 and Capan-2 cells incubated with FTS NCs or FTS/YM155 NCs (0.04, 0.08, 0.17, 0.25, and 0.33 μM) for 24 and 48 h. (b) IC_{50} values of FTS NCs and FTS/YM155 NCs on A549 and Capan-2 cells following 24 and 48 h. Data were expressed as means ± SD (n = 3). **P* < 0.05, ***P* < 0.01, and ****P* < 0.001.

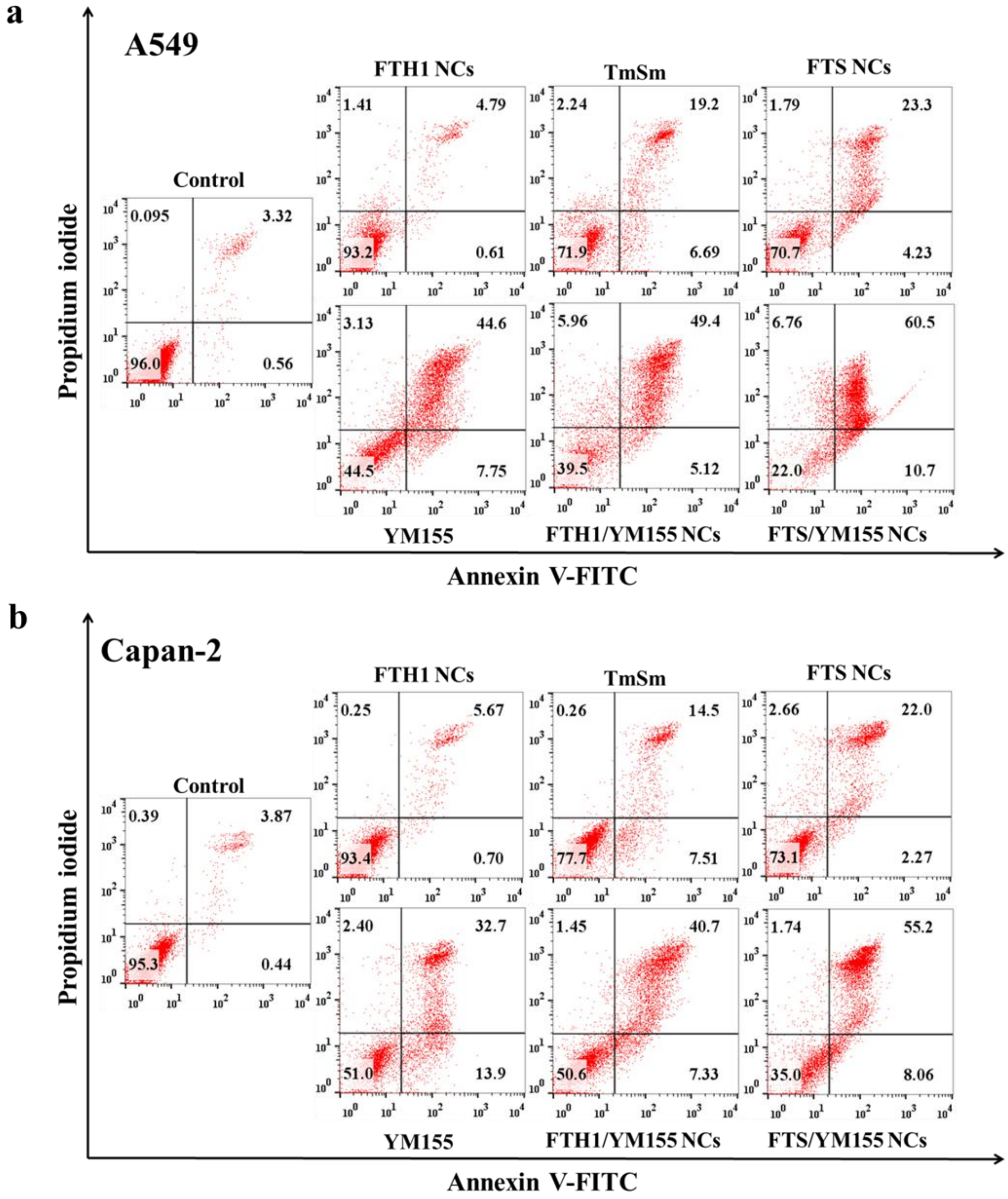

Figure S9. Apoptosis assay. Apoptosis induced by TmSm, YM155, FTH1 NCs, FTS NCs, FTH1/YM155 NCs, and FTS/YM155 NCs in (a) A549 and (b) Capan-2 cells for 48 h detected by the Annexin V-FITC staining test using flow cytometry. The final concentrations of TmSm and YM155 were kept at 2 and 0.15 μM, respectively.

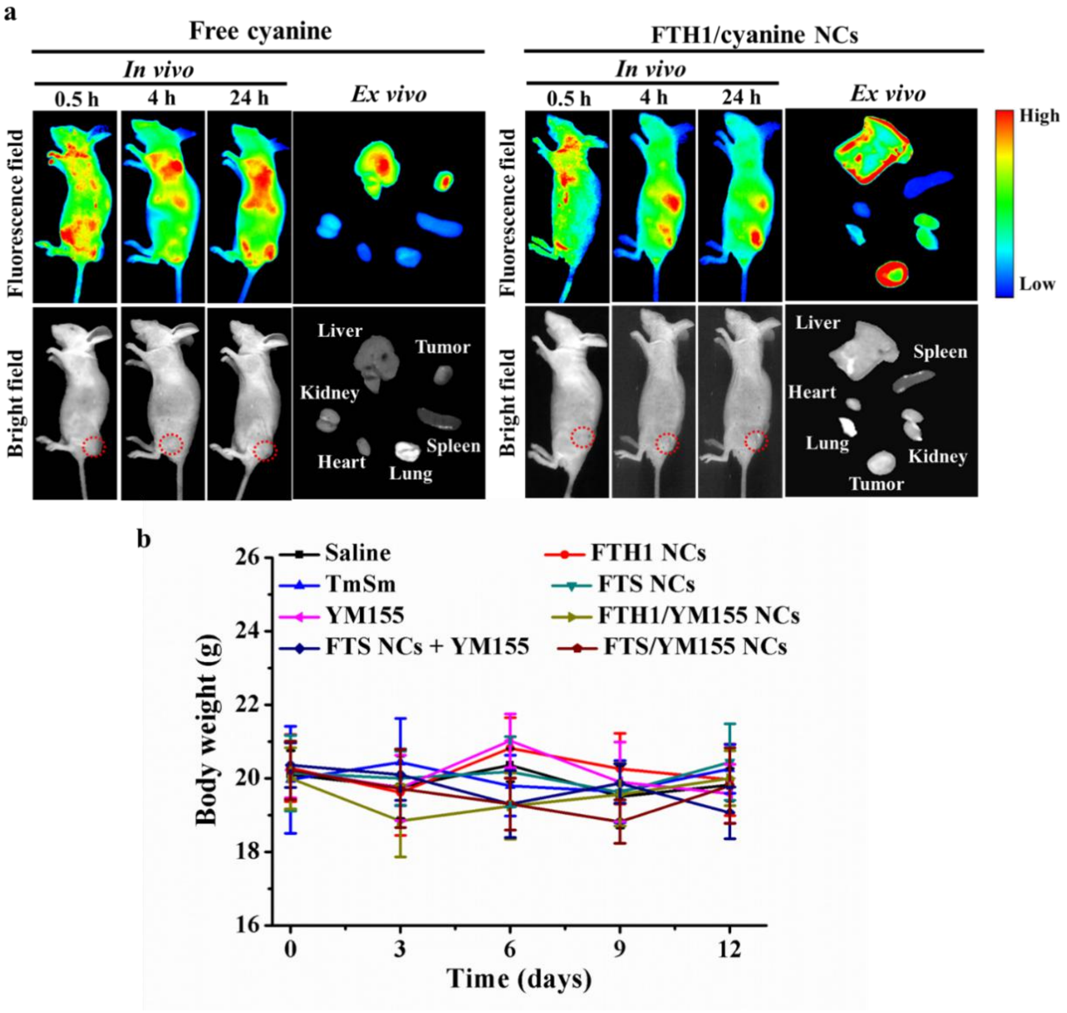

Figure S10. *In vivo* imaging and body weight changes of A549 tumor-bearing mice. (a) *In vivo* imaging of subcutaneous tumor-bearing nude mice after intravenous injection of free cyanine and FTH1/cyanine NCs (cyanine, ex/em = 740/830 nm) at 0.5, 4, and 24 h, respectively. *Ex vivo* fluorescence images of major organs and tumors at 24 h post-injection. Red circle marks the tumor location. (b) The body weight of the mice was monitored every 3 days for 12 days. Data were expressed as means ± SD (n = 5).

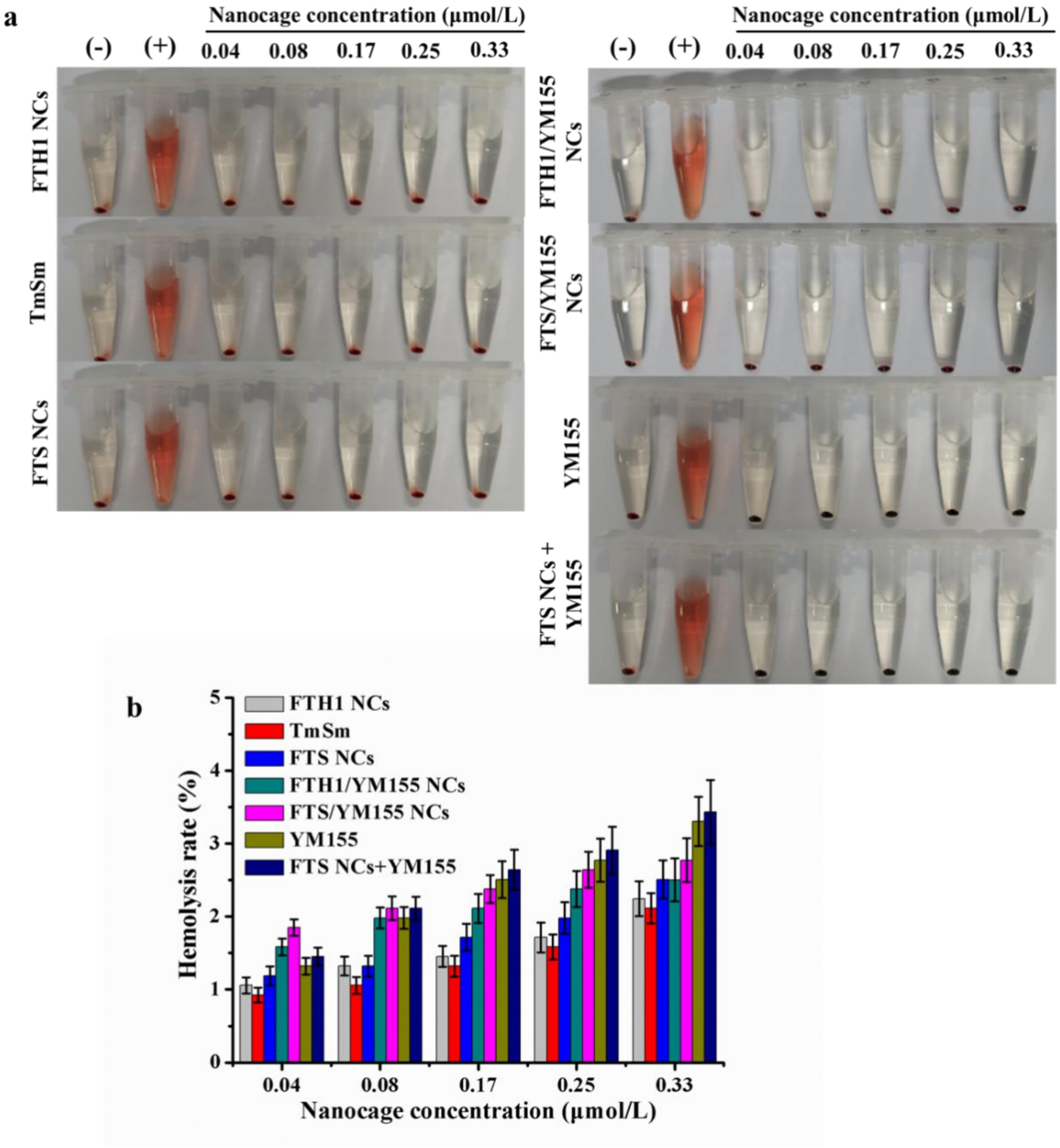

Figure S11. Hemolysis assay. (a) Images of blood of mice incubated with a series of concentration of YM155, TmSm, FTH1 NCs, FTS NCs, FTH1/YM155 NCs, FTS/YM155 NCs, the mixture of FTS NCs and YM155 for 3 h at 37 °C. Ultrapure water (+) and PBS (-) as positive and negative controls, respectively. (b) Corresponding quantification of hemolysis percent. Data were expressed as means ± SD (n = 3).

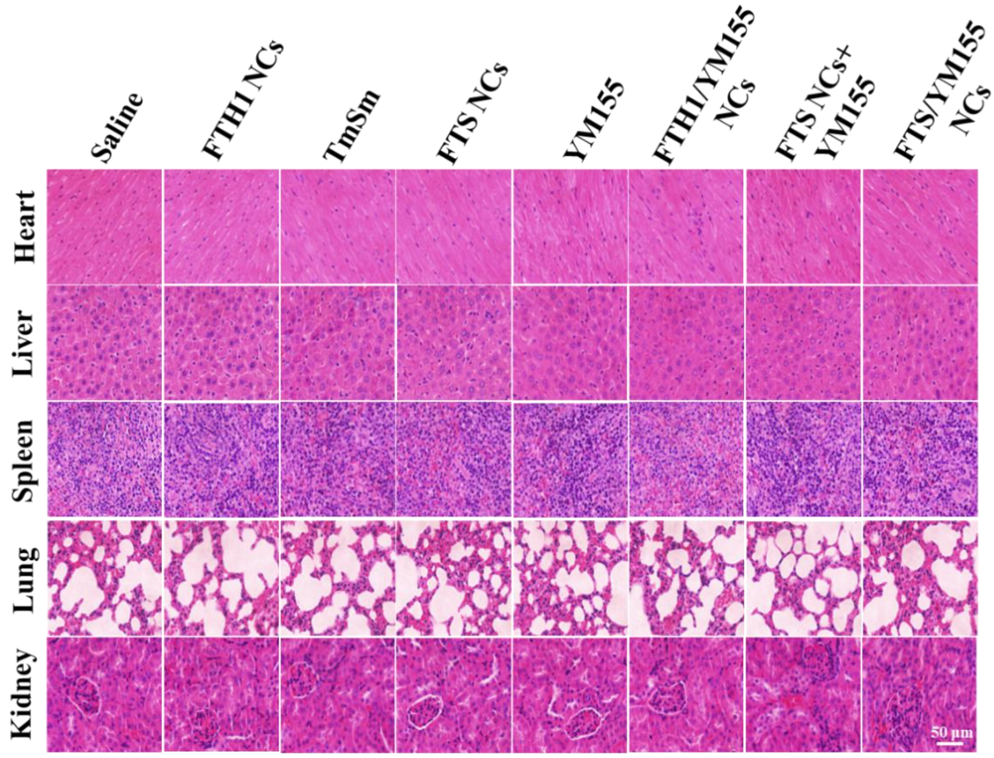

Figure S12. Histological examination of major organs. Fifteen days after treatment with YM155, TmSm, FTH1 NCs, FTS NCs, FTH1/YM155 NCs, FTS/YM155 NCs, the mixture of FTS NCs and YM155 in A549 tumor-bearing mice, mice were sacrificed. Major organs (heart, liver, spleen, lung, and kidney) were collected and fixed with 4% paraformaldehyde for paraffin slicing. Organ injuries were evaluated with HE staining. All images were taken by fluorescence microscopy at 400× magnification (Scale bar = 50 μm).

Table S1.The primer sequence for qPCR.

Gene	Primer sequence (5' →3')	Length (bp)
β-actin	Forward: ATTGGCAATGAGCGGTTC<br>Reverse: GGATGCCACAGGACTCCAT	76
Survivin	Forward: AAGAACTGGCCCTTCTTGGA<br>Reverse: CAACCGGACGAATGCTTTT	185
Caspase-3	Forward: GACATGGCGTGTCATAAAAT<br>Reverse: GCATAAATTCAAGCTTGTCG	160

Table S2. Comparison of tumor weight and tumor inhibition rate among different treated groups.

Groups	Tumor weight (g)	Tumor inhibition rate (%)
Saline	1.97 ± 0.14	/
FTH1 NCs	1.92 ± 0.10	3.85 ± 1.19
TmSm	1.19 ± 0.13	40.75 ± 3.01
FTS NCs	0.77 ± 0.05	62.35 ± 6.88[d]
YM155	1.85 ± 0.09	8.13 ± 1.00
FTH1/YM155 NCs	0.91 ± 0.07	54.86 ± 5.82
FTS NCs+YM155	0.67 ± 0.06	66.54 ± 6.55
FTS/YM155 NCs	0.23 ± 0.01	88.86 ± 5.94[a,b,c]

Data were expressed as means ± SD (n = 5). [a]$P < 0.05$ compared with FTS NCs, FTH1/YM155 NCs, and FTS NCs+YM155 group; [b]$P < 0.01$ and [d]$P < 0.05$ in comparison to TmSm group; [c]$P < 0.001$ compared with YM155 group.
